# Ubiquitin-specific peptidase 5 facilitates cancer stem cell-like properties in lung cancer by deubiquitinating β-catenin

**DOI:** 10.1186/s12935-023-03059-6

**Published:** 2023-09-19

**Authors:** Chia-Hao Tung, Jia-En Wu, Meng-Fan Huang, Wen-Lung Wang, Yi-Ying Wu, Yao-Tsung Tsai, Xiu-Rui Hsu, Sheng-Hsiang Lin, Yuh-Ling Chen, Tse-Ming Hong

**Affiliations:** 1 Institute of Clinical Medicine, College of Medicine, National Cheng Kung University, No. 1, University Road, Tainan, 70101 Taiwan; 2 Department of Internal Medicine, College of Medicine, National Cheng Kung University, Tainan, Taiwan; 3 Clinical Medicine Research Center, College of Medicine, National Cheng Kung University Hospital, National Cheng Kung University, Tainan, Taiwan; 4 Institute of Oral Medicine, College of Medicine, National Cheng Kung University, Tainan, Taiwan; 5 Biostatistics Consulting Center, College of Medicine, National Cheng Kung University Hospital, National Cheng Kung University, Tainan, Taiwan

**Keywords:** Cancer stem cells, USP5, β-catenin, Lung cancer, Metastasis

## Abstract

**Background:**

Lung cancer has the highest mortality rate in the world, and mounting evidence suggests that cancer stem cells (CSCs) are associated with poor prognosis, recurrence, and metastasis of lung cancer. It is urgent to identify new biomarkers and therapeutic targets for targeting lung CSCs.

**Methods:**

We computed the single-sample gene set enrichment analysis (ssGSEA) of 1554 Reactome gene sets to identify the mRNA expression-based stemness index (mRNAsi)-associated pathways using the genome-wide RNA sequencing data of 509 patients from The Cancer Genome Atlas (TCGA) cohort of lung adenocarcinoma (LUAD). Phenotypic effects of ubiquitin-specific peptidase 5 (USP5) on the CSC-like properties and metastasis were examined by in vitro sphere formation assay, migration assay, invasion assay, and in vivo xenografted animal models. Cycloheximide chase assay, co-immunoprecipitation assay, and deubiquitination assay were performed to confirm the effect of USP5 on the deubiquitination of β-catenin.

**Results:**

We demonstrated that *USP5* expression were positively correlated with the stemness-associated signatures and poor outcomes in lung cancer specimens. Silencing of endogenous USP5 reduced CSC-like characteristics, epithelial-mesenchymal transition (EMT), and metastasis in vitro and in vivo. Furthermore, USP5 interacted with β-catenin, which resulted in deubiquitination, stabilization of β-catenin, and activation of Wnt/β-catenin pathway. Accordingly, expression of USP5 was positively correlated with the enrichment score of the Wnt/TCF pathway signature in human lung cancer. Silencing of β-catenin expression suppressed USP5-enhancing sphere formation. Targeting USP5 with the small molecule WP1130 promoted the degradation of β-catenin, and showed great inhibitory effects on sphere formation, migration, and invasion. Finally, we identified a poor-prognosis subset of tumors characterized by high levels of *USP5*, Wnt signaling score, and Stemness score in both TCGA-LUAD and Rousseaux_2013 datasets.

**Conclusions:**

These findings reveal a clinical evidence for USP5-enhanced Wnt/β-catenin signaling in promoting lung cancer stemness and metastasis, implying that targeting USP5 could provide beneficial effects to improve lung cancer therapeutics.

**Supplementary Information:**

The online version contains supplementary material available at 10.1186/s12935-023-03059-6.

## Background

Lung cancer remains the most common cancer worldwide and has the highest mortality rate [1, 2]. Since early-stage lung cancer is difficult to diagnose, more than 60% of patients are diagnosed with advanced-stage disease, and the 5-year overall survival rate is approximately 15% [3]. Metastasis is the greatest challenge in the treatment of lung cancer [4]. Moreover, the prognosis of lung cancer patients is poor, and the 5-year overall survival rate is low [5]. Recently, a breakthrough in immunotherapy for lung cancer patients with immunodeficiency, who are observed to have high expression of PD-1/PD‐L1 and suppression of T cells, was made [6]. Overall, despite the encouraging development in immunotherapy, improvements in the therapeutic approaches for patients with lung cancer are still needed.

Mounting evidence shows that cancer stem cells (CSCs) are responsible for metastasis, tumor growth and therapeutic resistance [7]. Similar to somatic stem cells, tumor cells with CSC properties, which account for only a only small proportion of all tumor cells, have a great self-renewal capacity and the abilities to differentiate and generate cells with tumorigenic and nontumorigenic properties, which are important for establishing and maintaining tumors [8]. The CSC properties of lung cancer can be characterized by several methods and include tumor sphere-forming ability, stem cell marker expression, tumorigenic potential, increased invasiveness, and apoptosis resistance [9]. However, the key factors that control the stemness properties of lung cancer remain largely unclear.

Protein ubiquitination is a reversible process [10, 11]. Deubiquitinating enzymes (DUBs) are able to remove ubiquitin from target proteins to regulate the activities of substrates [12, 13]. DUBs reverse the actions of E3 ligases by cleaving the isopeptide bond between target proteins and ubiquitin [14]. At present, there are approximately 100 different DUBs identified in humans [15]. Recently, several studies have revealed that DUBs regulate many cellular functions and are closely linked to parameters of tumor progression, such as tumor growth, metastasis, and drug resistance [16]. However, little is known about the relationship between DUBs and CSCs.

To comprehensively investigate the involvement of signaling pathways in CSCs, we first analyzed the correlation between reactome pathway enrichment scores and stemness-related scores, the mRNA expression-based stemness index (mRNAsi) from Malta’s study [17], in The Cancer Genome Atlas (TCGA)-lung adenocarcinoma (LUAD) dataset. Interestingly, DUB-related pathways were shown to be highly correlated with both the stemness index and poor overall survival in lung cancer patients. Moreover, we found ubiquitin-specific peptidase 5 (USP5) to be the most significant candidate correlated with both the mRNAsi and poor overall survival in lung cancer. The data support an association between USP5 and CSCs in lung cancer. However, the functional role of USP5 in the regulation of CSCs and the underlying mechanisms remain elusive. In this study, we focused on exploring the roles of USP5 in regulating lung CSCs and the clinical impacts.

## Materials and methods

### Analysis of mRNAsi-associated reactome pathways and DUBs in TCGA-LUAD dataset

Publicly available TCGA data on LUAD were obtained from the UCSC Xena browser (https://xenabrowser.net/datapages/). The GDC HTSeq FPKM RNAseq dataset (version 07-20-2019) and survival data were downloaded for analysis. For RNA-seq data, Fragments Per Kilobase of transcript per Million mapped reads (FPKM) was used to represent the expression levels of genes. To study the correlation between mRNAsi and reactome pathway activities, the mRNAsi of each TCGA-LUAD sample was downloaded from the attachment for Malta’s study [17]. We merged the miRNAsi index with RNA-seq data, and three unmatched cases were deleted. A total of 509 TCGA-LUAD samples were used for the further single-sample gene set enrichment analysis (ssGSEA) to calculate the relative Reactome pathway activities (MSigDB, version 7.1 - www.broadinstitute.org/gsea/msigdb) [18, 19]. The ssGSEA was performed by *R/Bioconductor* package GSVA (v1.34.0) [20]. The correlation between miRNAsi index and pathway activities was determined using Pearson’s correlation analysis. Focusing on the Reactome pathways which were positively correlated with miRNAsi index (Pearson *r* > 0.7), individual ssGSEA scores were plotted as a heatmap with the *R* package pheatmap (version 1.0.12).

For survival analysis, a total of 500 LUAD patients with fully transcriptomic profile and intact prognosis information were used. Survival times were censored after 5 years to reduce the confounding effect of patient age. The relationship between the ssGSEA score of top 20 pathways and patient overall survival was explored through a univariate Cox model using the coxph() function in the *R* package survival (v3.1-12).

For identifying the potential DUBs associated with mRNAsi and poor overall survival in the gene set REACTOME_UB_SPECIFIC_PROCESSING_PROTEASES, GSEA was performed [18]. The seven ‘leading edge’ genes with DUB enzyme activities were selected for further survival analysis. Patients were classified according to the mean expression of DUBs into high or low expression groups. Univariate Cox regression analysis was used to identify the DUBs with prognostic value.

### Cell lines and reagents

The lung cancer cell lines CL1-5 cultured in RPMI 1640 media supplemented with 10% fetal bovine serum (FBS, Gibco, Grand Island, NY) were established and characterized as previously described [21]. Lung cancer cell line A549, H23, H1299, and H522 were purchased from the American Type Culture Collection (ATCC). LIJ, a primary cell line from a non-small-cell lung cancer (NSCLC) patient, were kindly provided by Dr. Wu-Chou Su (Institute of Molecular Medicine, National Cheng Kung University, Taiwan). A549 and H1299 cells were culture in Dulbecco’s Modified Eagle Medium (Invitrogen) containing 10% FBS. The other cell lines were all maintained in RPMI-1640 media (Invitrogen) supplemented with 10% fetal bovine serum. All of the cell lines were maintained at 37^o^C in a humidified atmosphere with 5% CO_2_. ATCC cell lines were authenticated using short tandem repeat analysis, and all cell lines were tested for free of mycoplasma contamination. WP1130 (Degrasyn) and Vialinin A were purchased from Selleck Chemicals (#S2243) and Tocris Bioscience (#4988), respectively.

### Western blotting

Proteins were fractionated on an SDS-PAGE gel and transferred to an Immobilon-P PVDF membrane (0.45 μm, Millipore). After blocking, membranes were incubated with primary antibodies and then anti-rabbit or anti-mouse secondary antibodies were used for detection. Immunoreactive proteins were visualized using Western Lightening Plus-ECL (PerkinElmer, #NEL105001EA). The following primary antibodies were used: anti-β-actin monoclonal antibody (Sigma, #A5441); anti-USP5 monoclonal antibody (Santa Cruz, #sc-390,943), anti-β-catenin monoclonal antibody (BD Biosciences, #610,153), anti-Slug polyclonal antibody (Santa Cruz, #sc-10,436), anti-E-cadherin monoclonal antibody (BD Biosciences, #610,182), anti-N-cadherin monoclonal antibody (BD Biosciences, #610,921), anti-Vimentin monoclonal antibody (Cell Signaling Technology, #5741S), and anti-Ubiquitin monoclonal antibody (Santa Cruz, #sc-8017).

### RNA isolation and quantitative real-time PCR (qRT-PCR)

RNA was extracted using the TRIzol Reagent (Invitrogen), and RNA was reverse transcribed with the ImProm-II Reverse Transcription System (Promega). qRT-PCR was performed using the Fast SYBR Green Master Mix (Applied Biosystems) by the StepOne Real-Time PCR Systems (Applied Biosystems). Each sample was analyzed in triplicate and *TBP* was used as an internal control. The data were analyzed by the StepOne software v2.3 (Applied Biosystems). The sequences of various PCR primers were listed in Additional file 1: Table S1.

### Lentivirus transduction

Lentivirus-based shRNA constructs targeting *USP5* (TRCN0000004069 for shUSP5#1 and TRCN0000004070 for shUSP5#2) and *CTNNB1* (TRCN0000003843 for shCTNNB1#1 and TRCN0000003845 for shCTNNB1#2) were obtained from the Taiwan National RNAi Core Facility. The shRNA targeting LacZ (TRCN0000072224) was used as negative control. The full-length USP5 ORF was cloned into the BamHI/XhoI sites of the pLEX lentiviral vector (Thermo Scientific). Lentivirus was produced by cotransfection of either pLEX or pLKO-shRNA constrcuts with the package plasmids (pCMV-ΔR8.91 and pMD.G) into HEK293T cells using Lipofectamine 2000 (Invitrogen). The culture supernatants containing viral particles were collected at 24 and 48 h post-transfection. For establishing shRNA stable cell lines, 48-hour post-infected cells were treated with puromycin for selecting a pool of stable clones.

### Sphere formation assay

For sphere formation assay, lung cancer cells were plated in poly-2-hydroxyethyl methacrylate (poly-HEMA, Sigma-Aldrich)-coated cell culture dishes (0.8 mg/cm^2^) to prevent cell adhesion and grown in a serum-free DMEM-F12 medium containing 0.5% methylcellulose (Sigma-Aldrich), supplemented with N2 supplement (Invitrogen), 20 ng/ml of EGF (Peprotech) and 20 ng/ml of bFGF (Peprotech). After 14 days of culture, plates were scanned and stitched brightfield images were produced using NIS-Elements software (Nikon). The number of spheres ( ≧ 75 μm) were counted.

### In vitro Limiting dilution analysis (LDA)

For in vitro LDA analysis [22], indicated cell doses of lung cancer cells were plated in 96-well plates coated with poly-HEMA (Sigma-Aldrich) at the density of 0.8 mg/cm^2^. After 14 days, wells containing spheres were measured under microscope. The number of positive wells was used to calculate the sphere-initiating cell frequency by the extreme limiting dilution analysis (ELDA) software (http://bioinf.wehi.edu.au/software/elda/index.html) [23].

### Flow cytometric analysis

Cells were dissociated using Cellstripper (Corning, #25-056-CI). Cells were stained with PE-conjugated mouse anti-human CD44 antibody (Southern Biotech, #9400-09) at 4 °C for 30 min. Data were collected using a FACS Calibur flow cytometer (BD Biosciences).

### In vitro wound healing assays

In vitro wound healing assays were performed by inoculating lung cancer cells in Culture-Inserts (Ibidi). Briefly, 2 × 10^4^ cells were seeded into each well of inserts in 3.5 mm dishes. After cell attachment for 16 h, the inserts were removed. Cells migrated into the wound area were captured by microscope and measured for each indicated time by ImageJ software.

### Transwell invasion assay

24-well polycarbonate Transwell filters (pore size 8 μm; Corning Costar) coated with 30 µg of Matrigel (BD Biosciences) were used for cell invasion assays. Briefly, 1 × 10^5^ CL1-5 and A549 cells were suspended in serum free medium and plated in the upper chamber, medium containing 10% FBS was added in the lower chamber. After 24-hour culture, the invaded cells were counted. On the upper surface of the filter, non-penetrating cells were removed with a cotton swab. Penetrating cells were stained by Liu’s stain (ASK). The number of cells invaded into the lower surface Cells were counted under an inverted microscope.

### Animal experiments

For the in vivo LDA, a limiting dilution series of cells harboring shLacZ or shUSP5#2 was injected subcutaneously into the flanks of 6-week-old non-obese diabetic-severe combined immunodeficiency (NOD-SCID) mice (*n* = 6 per group). After 45 days, the number of mice with tumors in each group was quantified. The tumor initiating frequency was calculated by the ELDA software. For studying lung metastasis in mouse model, 1 × 10^6^ CL1-5-Luc cell line stably transfected with nonsilencing control shRNA or USP5 shRNA were subcutaneously implanted into the left posterior flank of each 6-week-old NOD-SCID mice. After 21 days, luciferase activity in the lungs were acquired using an IVIS Spectrum In Vivo Imaging System (PerkinElmer). Photon counts per area were compared with *t* test to calculate the *P* value. All mouse experiments were approved by the Institutional Animal Care and Use Committee (IACUC) of National Cheng Kung University, Tainan, Taiwan.

### Cycloheximide chase assay

The half-life of slug or β-catenin proteins were determined using cycloheximide inhibition of protein synthesis. Cells were plated into 6-well at 2 × 10^5^ cells per well and allowed to attach overnight. Next, cells were incubated with cycloheximide (100 µg/ml, Sigma-Aldrich) and harvested at each indicated time. Western blot analysis was performed by using the anti-β-catenin antibodies to determine the half-life of proteins.

### Immunoprecipitation assay

Protein extracts were prepared in IP buffer [0.5% Triton X-100, 137 mM NaCl, 19 mM Tris-HCl, 2.68 mM KCl, 30mM Na-Pyrophosphate, pH 7.4, 0.1mM Na_3_VO_4_, 50mM NaF and protease inhibitor cocktail (Roche)], follow by passing 5 times through a 21-gauge needle. After centrifugation, 1 mg of supernatants were incubated with 1 µg of anti-β-catenin antibody or Mouse IgG (Sigma-Aldrich) for overnight at 4 ℃. For precipitation, the immunocomplexes were incubated with protein G magnetic beads (Millipore) at 4 ℃ for 1 h. Protein complexes were eluted by heating at 95 °C for 5 min in loading buffer.

### Immunohistochemical staining

Tissues were fixed in 10% formalin, paraffin-embedded and sectioned. The 4-µm-thick sections were used for staining. Heat-induced antigen retrieval buffers were used (citrate buffer, PH = 6, for USP5 detection; and Tris-EDTA buffer, PH = 9, for β-catenin detection) and heated for 40 min. Primary antibodies of USP5 (Santa Cruz) and β-catenin (BD biosciences) were diluted at a 1:50 dilution, respectively. Biotin-labeled secondary antibody Trekkie Universal Link (Biocare Medical, USA, STU700H) was used. Streptavidin-HRP were used to detect the antigens and visualized by 3-amino-9-ethylcarbazole (AEC) Substrate-Chromogen (ScyTek Laboratories, Logan, UT, ACD015), followed by hematoxylin counterstain.

### Gene set enrichment analysis

For identifying the potential pathways associated with either mRNAsi or USP5 expression in TCGA-LUAD dataset, GSEA was conducted with 1,000 phenotype permutations using a continuous increasing phenotype label based on either mRNAsi or *USP5* expression. The metric for ranking genes was set as ‘Pearson’.

### Detection of ubiquitinated β-catenin

Cells in 10 cm dish were treated with 10 µM MG132 (Sigma-Aldrich, #7449) for 6 h and then lysed with RIPA lysis buffer, follow by passing 5 times through a 21-gauge needle. 1 mg of whole-cell extract was used for immunoprecipitation with 1 µg anti-β-catenin antibody (BD Biosciences, #610,153), and then ubiquitinated β-catenin was detected by western blotting using anti-Ubiquitin antibody.

### Cellular thermal shift assay

Engagement between WP1130 and USP5 in cells was analyzed by Cellular thermal shift assay (CETSA). For a CETSA in living CL1-5 cells, 1 × 10^6^ cells were seeded in 10-cm dishes and exposed to WP1130 (5 µM) or DMSO for 1 h. Following incubation, cells were harvested with PBS supplemented with complete protease inhibitor mixture. The cell lysates were then divided into smaller aliquots and heated in a thermal cycler (37–69 °C gradient) for 3 min. Cells were lysed by three freeze-thaw cycles in liquid nitrogen, and then centrifuged at 20,000 g for 20 min at 4 °C to separate the soluble fractions from the precipitates. The supernatant was analyzed by immunoblot using USP5 antibody after SDS-PAGE. CETSA curves were obtained by plotting percentage of soluble USP5 against temperature.

### Bioinformatic database analysis

The clinical annotated data were downloaded from the GEO database. GSE2514, GSE19188, and GSE41271 were used to examine the correlation of *USP5* in normal and tumor tissues. The correlations between *USP5* expression and overall survival of lung cancer patients were analyzed by 6 datasets, including TCGA_LUAD, Rousseaux_2013 (GSE30219), Schabath_2016 (GSE72094), Okayama_2012 (GSE31210), Wilkerson_2012 (GSE26939) and Raponi_2006 (GSE4573), downloaded from Lung Cancer Explorer (http://lce.biohpc.swmed.edu/lungcancer/) [24]. The optimal cut point for *USP5* expression was determined by using X-tile software version 3.6.1 [25]. GSE50081 and GSE17710 were used to determine the correlation between *USP5* and *SNAI2* expression.

### Statistical analysis

Statistical analyses were performed with GraphPad Prism 6.0. Results were presented as mean ± SD, median or percentage frequency. Differences between groups were determined by unpaired *t* test or Mann-Whitney *U* test. Normalized enrichment scores of GSVA were computed through setting “max.diff” to “TRUE,“. Pearson correlation coefficients were used to test correlations between each gene expression and ssGSEA scores in clinical specimens. Kaplan-Meier curves were performed to estimate survival rates and were compared by log-rank test. All results were considered significant if *P*-value was less than 0.05.

## Results

### *USP5* is identified to be a stemness-related biomarker for predicting poor outcomes in lung cancer

**Fig. 1:**
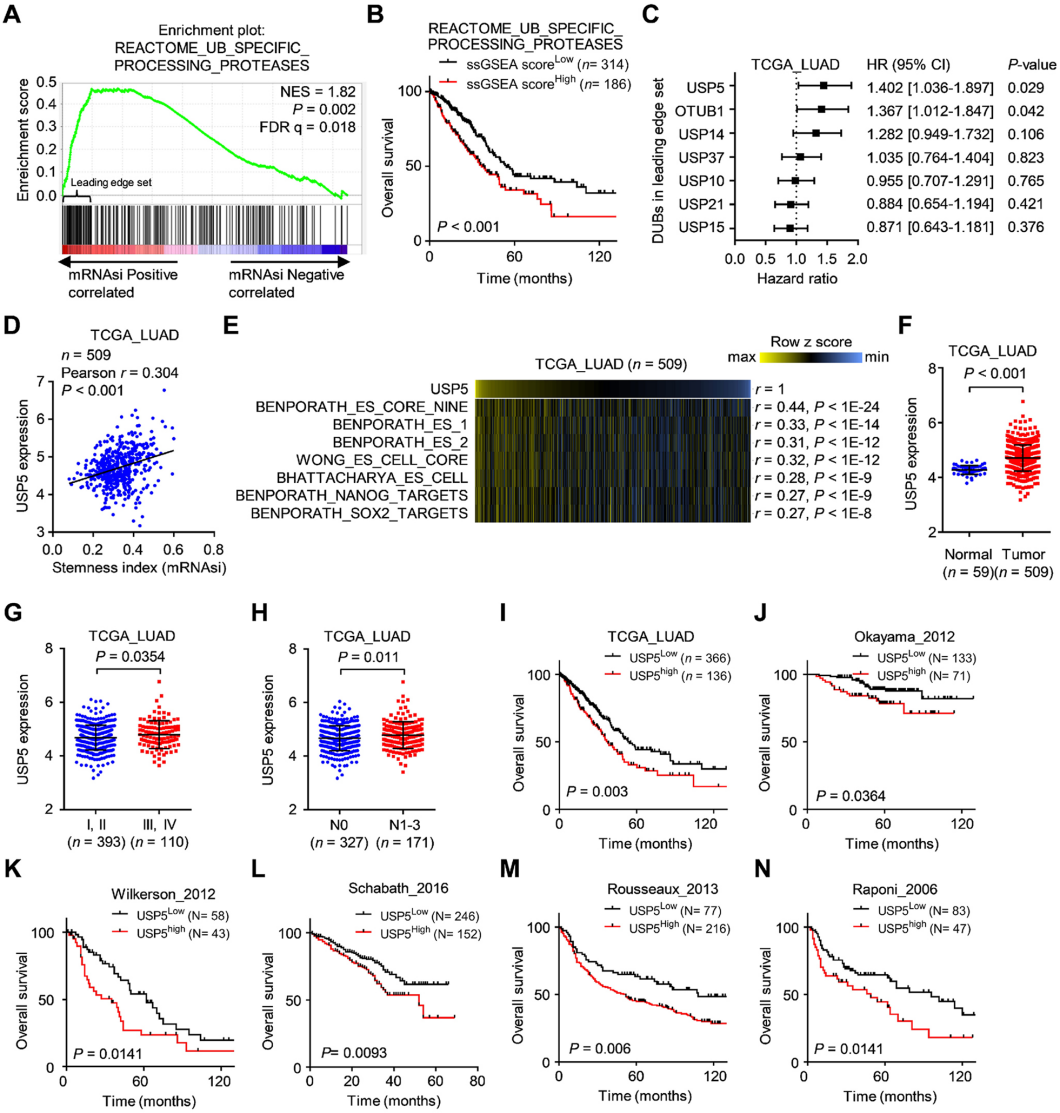
USP5 is significantly associated with cancer stemness and prognosis in lung cancer. **A** Gene set enrichment analysis (GSEA) enrichment plots of the gene sets from EACTOME_UB_SPECIFIC_PROCESSING_PROTEASES in the TCGA-LUAD dataset (*n* = 509) ranked by Pearson correlation results (Pearson *r*) for the stemness index (mRNAsi). **B** Kaplan-Meier analysis of TCGA lung cancer patients (*n* = 500) stratified by the scores for ssGSEA of deubiquitinases. **C** Univariate Cox regression analysis of 7 DUBs in the ‘leading edge’ genes (related to **A**) for overall survival in TCGA-LUAD dataset. HR, hazard ratios; CI, confidence intervals. **D** Positive correlation between *USP5* expression and the mRNAsi in the TCGA-LUAD dataset. *r*, Pearson correlation coefficient. **E** Heatmap showing positive correlation between *USP5* expression and ssGSEA scores for stemness-related gene signatures from the TCGA-LUAD dataset. **F** The expression levels of *USP5* in lung tumor samples (*n* = 509) were significantly higher than those in normal lung tissue samples (*n* = 59). **G** The expression levels of *USP5* were significantly higher in late-stage tumors (stage III or IV; *n* = 110) than in early-stage tumors (stage I or II; *n* = 393) in the TCGA-LUAD dataset. **H ***USP5* expression in lung cancer patients with lymph node metastasis (N1-N3; *n* = 171) was significantly higher than that in those without metastasis (N0; *n* = 327) in the TCGA-LUAD dataset. **I–N** Kaplan-Meier curves showed significantly poorer overall survival in the high *USP5* expression subgroup than in the low *USP5* expression subgroup in the TCGA-LUAD (**I**), Okayama (**J**), Wilkerson (**K**), Schabath (**L**), Rousseaux (**M**) and Raponi (**N**) cohorts. *P*-values were based on log-rank tests

CSCs have been identified as important factors affecting recurrence and progression in NSCLC [8]. Recently, Malta’s study identified the mRNAsi, which has been used to quantify the stemness status of clinical specimens [17]. Higher values for mRNAsi are positively correlated with active biological processes related to stemness and tumor dedifferentiation [17]. To identify a potential pathway contributing to CSCs in lung cancer, we obtained the mRNAsi of the TCGA-LUAD samples from Malta’s study [17]. Next, we downloaded the global transcriptomic profile of TCGA-LUAD samples and used ssGSEA to determine the reactome pathway intensities of each sample. Based on the 135 pathways positively correlated with the mRNAsi (Pearson r > 0.7), we used univariate Cox regression analyses to identify the top 10 pathways significantly correlated with poor overall survival in lung cancer patients using the TCGA-LUAD dataset (Additional file 1: Figure S1A, B). Seven of the top 10 pathways were related to the regulation of the cell cycle/mitosis (including the top 2 pathways) (Additional file 1: Figure S1B), which concurs with similar results reported in a recent study showing that cell cycle-related pathways are associated with CSC features and poor overall survival in LUAD [26]. Intriguingly, we found that the 3rd highest ranked pathway, REACTOME_UB_SPECIFIC_PROCESSING_ PROTEASES, was related to protein deubiquitination, suggesting that the protein deubiquitination process is highly correlated with stemness and survival in lung cancer. These results were further confirmed by GSEA and Kaplan-Meier survival analysis (Fig. 1A, B). Since the relationship between deubiquitination and lung CSCs remains largely unclear, we focused on identifying proteins with deubiquitinase activity related to lung CSCs. To achieve this, we analyzed the GSEA results to obtain the genes in the leading-edge subset that were highly correlated with the mRNAsi. Focusing on seven genes with deubiquitinase activity, we found that USP5 was the most significant candidate associated with poor overall survival in patients with lung cancer (Fig. 1C). Furthermore, USP5 expression was shown to be positively correlated with mRNAsi, suggesting a connection between USP5 and CSCs in lung cancer (Fig. 1D and Additional file 1: Figure S2A). To further understand the relationships between USP5 and stemness properties, we performed ssGSEA to test the correlations between USP5 and stemness-related pathways [19, 27] in the TCGA-LUAD dataset. Indeed, several stemness-associated signatures were found to be positively correlated with USP5 expression in the TCGA-LUAD dataset (Fig. 1E). Overall, our data suggest that USP5 is a potential target that is highly correlated with stemness activities in lung cancer.

While trying to comprehensively understand the association between USP5 and the clinical outcomes of lung cancer patients, we first found significantly increased expression of USP5 in human LUAD samples (*n* = 509) compared to normal tissue samples (*n* = 59) in the TCGA-LUAD dataset (Fig. 1F). Similar results were also found in two other datasets, GSE19188 and GSE2514 (Additional file 1: Figure S2B, C). In addition, the expression of USP5 was significantly higher in late-stage tumors (III + IV, *n* = 110) than in early-stage tumors (I + II, *n* = 393) in the TCGA-LUAD (Fig. 1G) and GSE3141 datasets (Additional file 1: Figure S2D). Furthermore, the expression of USP5 was significantly higher in tumors with lymph node metastasis than in those without metastasis in the TCGA-LUAD and GSE50081 datasets (Fig. 1H and Additional file 1: Figure S2E). In an investigation of the association between USP5 and survival in patients with lung cancer, we found that high USP5 expression was significantly correlated with poor overall survival in 6 independent GEO datasets, including TCGA_LUAD (Fig. 1I), Okayama_2012 (Fig. 1J), Wilkweson_2012 (Fig. 1K), Schabath_2016 (Fig. 1L) Rousseaux_2013 (Fig. 1M), and Raponi_2006 (Fig. 1N). Taken together, our results strongly suggest that USP5 expression is associated with cancer stemness and poor clinical outcomes in patients with lung cancer.

### Knockdown of *USP5* suppresses characteristics of lung CSCs

**Fig. 2:**
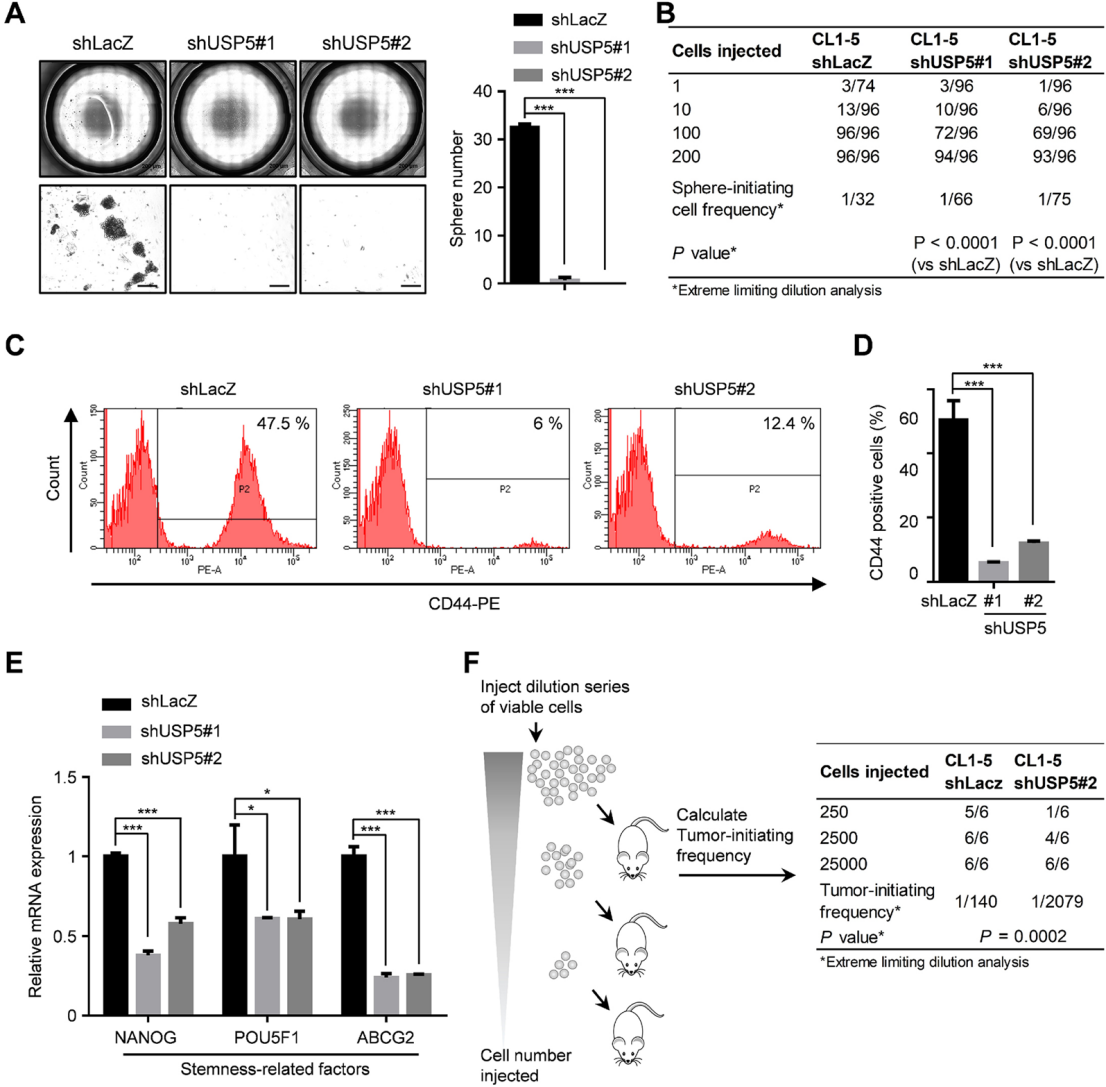
Knockdown of USP5 expression suppresses stemness properties in lung cancer. **A** CL1-5-shLacZ, shUSP5#1, and shUSP5#2 cells were cultured at 1,000 cells per well in low-attachment plates to assess sphere formation. Left panel, representative stitched brightfield images were produced using NIS-Elements software (Nikon). Scale bar: 200 μm. Right panel, quantification of spheres generated by CL1-5-shLacZ, shUSP5#1 or shUSP5#2 cells after 14 days. **B** shLacZ-, shUSP5#1-, and shUSP5#2-transduced cells were dissociated into single cells, and viable cells were plated in low-attachment 96-well plates for a limiting-dilution assay. The percentage of wells with spheres was measured and used to compute the limiting dilution to determine the frequency of sphere-initiating cells. The frequency and *P*-value were calculated using ELDA software. **C **and **D** Representative flow cytometric analysis **(C)** and quantification **(D)** of CD44^+^ subpopulations of shLacZ, shUSP5#1 and shUSP5#2 cells. **E** The mRNA levels of stemness-related factors in CL1-5-shLacZ, shUSP5#1 and shUSP5#2 cells. **F** CL1-5-shLacZ and shUSP5#2 cells (25,000, 2,500 or 250 cells) were resuspended in a Matrigel solution (1:1 Matrigel:PBS) and subcutaneously injected into NOD/SCID mice. Tumor incidence was assessed for each dilution of cells for 8 weeks, and the CSC frequency was calculated using ELDA software. In all bar plots, *P < 0.05 and ***P < 0.001 by a two-tailed Student’s *t* test

The above results indicated an association between USP5 and the mRNAsi in clinical specimens of lung cancer. However, the role of USP5 in lung CSCs remained unclear. To explore this, we next investigated whether USP5 can promote stemness properties in lung cancer. Using a sphere formation assay and an in vitro LDA [22], our data showed that compared to control CL1-5-shLacZ cells, USP5-knockdown CL1-5 cells showed a significantly suppressed sphere formation efficiency, as reflected by reduced numbers of spheres (Fig. 2A) and a reduced frequency of sphere-initiating cells (Fig. 2B). Moreover, overexpression of USP5 promotes sphere-forming ability in A549 cells (Additional file 1: Figure S3). Abundant evidence confirms that CD44 functions as a CSC surface marker in lung cancer and regulates several important properties related to cancer stemness, including self-renewal, tumor initiation, and metastasis [28, 29]. Moreover, several stem cell factors are reported to be important CSC markers in lung cancer, such as octamer-binding transcription factor 4 (Oct4, encoded by the *POU5F1* gene), Nanog homeobox (Nanog, encoded by the *NANOG* gene) and ABCG2 [28, 30]. To examine whether USP5 affects CD44 expression, we performed flow cytometric analysis to measure membrane CD44 expression. The proportion of CD44-positive cells was significantly decreased after knockdown of USP5 expression (Fig. 2C, D). In addition, our results further showed that silencing USP5 significantly reduced the mRNA expression of *NANOG*, *POU5F1*, and *ABCG2* in CL1-5 cells (Fig. 2E). To assess the role of USP5 in regulating tumor-initiating potential, a limiting-dilution tumorigenesis experiment, which remains the gold standard for identifying CSCs [31], was performed in vivo. Indeed, knockdown of USP5 expression significantly reduced the tumor-initiating cell frequency by 14.85-fold (Fig. 2F). Collectively, these results show that USP5 plays an important role in maintaining stemness properties in lung cancer.

#### USP5 drives EMT and metastasis in lung cancer

**Fig. 3:**
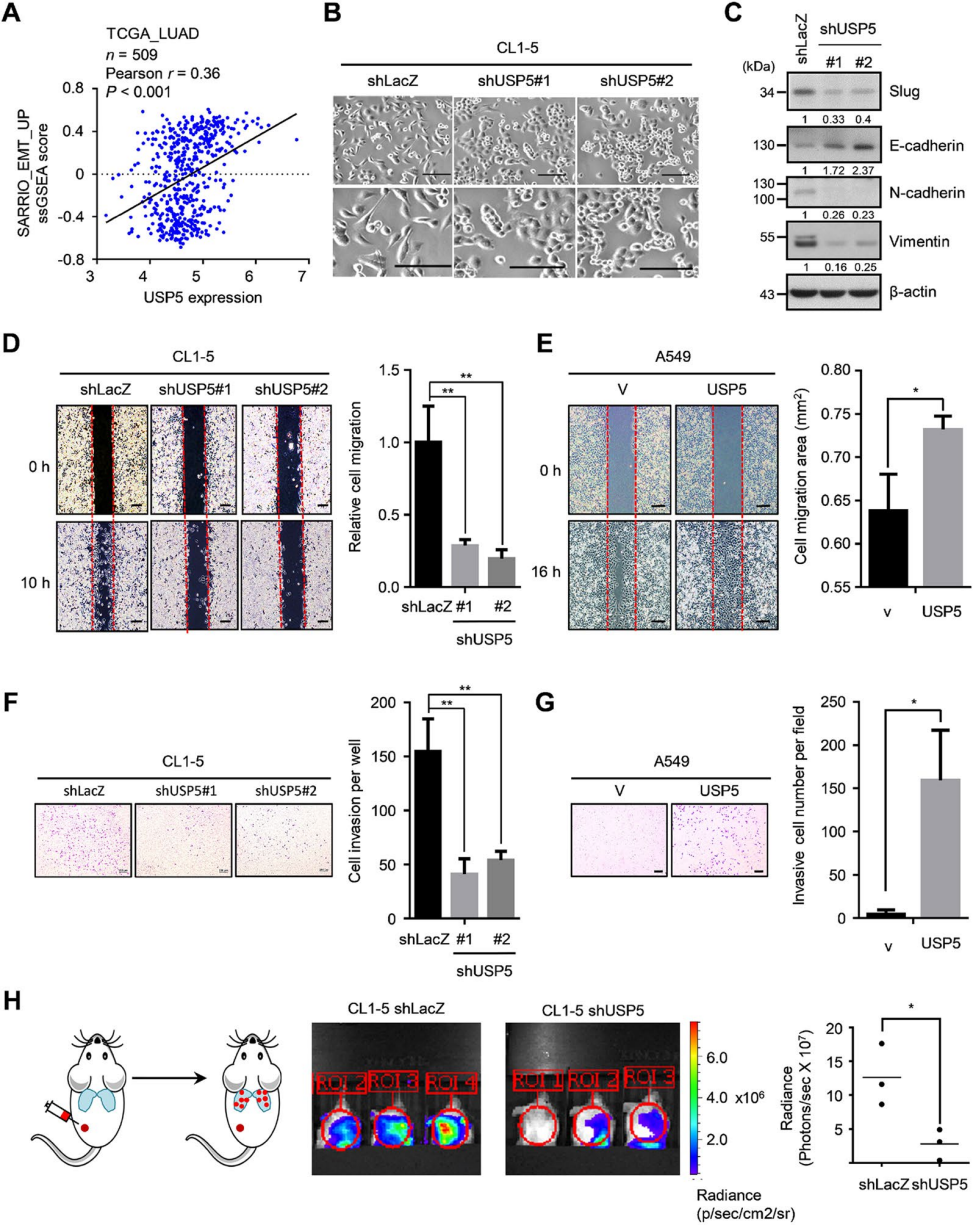
USP5 drives EMT and metastasis in lung cancer. **A** Significant correlation between USP5 expression and the ssGSEA score of the SARRIO_EMT_UP gene set in the TCGA-LUAD dataset. *r*, Pearson correlation coefficient. **B** Representative images of CL1-5-shLacZ, shUSP5#1 and shUSP5#2 cells. Scale bar, 100 μm. **C** Protein levels of Slug, E-cadherin, N-cadherin, and Vimentin in CL1-5-shLacZ, shUSP5#1 and shUSP5#2 cells. β-actin was used as an internal control. **D** Wound healing analysis of CL1-5-shLacZ, shUSP5#1, and shUSP5#2 cells at 10 h. Scale bar: 200 μm. **E** Wound healing analysis of A549 cells stably expressing either vector or USP5 at 16 h. Scale bar: 200 μm. **F** Transwell invasion analysis of CL1-5-shLacZ, shUSP5#1, and shUSP5#2 cells. Scale bar: 200 μm. **G** Transwell invasion analysis of A549 cells stably expressing either vector or USP5. Scale bar: 200 μm. **H **In vivo bioluminescence images of NOD/SCID mice subcutaneously injected with Luc-CL1-5 cells expressing either shLacZ or shUSP5#2 at 21 days after injection (right). Photon counts per area were measured. In all plots, *P < 0.05 and **P < 0.01 by a two-tailed Student’s *t* test

Emerging studies highlight the close relationship between EMT and CSC formation [32]. The prevailing theory states that CSCs often exhibit EMT properties [33]. Likewise, a recent comprehensive study also indicated that lung cancer metastasis was highly associated with the upregulation of an adult stem cell signature [34]. These findings were consistent with our analysis, which showed that the mRNAsi was positively correlated with an EMT-upregulation signature in the TCGA-LUAD dataset, indicating a connection between stemness and the EMT phenotype in lung cancer (Additional file 1: Figure S4A). However, the relationship between USP5 and EMT remained largely unknown in lung cancer. The results of ssGSEA revealed that USP5 expression was positively correlated with the EMT-upregulation signature in the TCGA-LUAD dataset, indicating that USP5 may be involved in the regulation of EMT (Fig. 3A). Furthermore, we found that USP5-silenced cells underwent a change in phenotype into an epithelial-like morphology and appeared round compared to control cells (Fig. 3B). We observed that USP5 silencing caused the induction of the epithelial marker E-cadherin and diminished the expression of the mesenchymal markers, vimentin and N-cadherin (Fig. 3C). In regard to EMT transcription factors, we found that knockdown of USP5 expression caused reductions in both the mRNA and protein (Slug) expression of *SNAI2* (Fig. 3C and Additional file 1: Figure S4B). In addition, this connection could also be observed in clinical specimens, which showed that *USP5* was positively correlated with *SNAI2* expression in two independent lung cancer datasets (Additional file 1: Figure S4C, D). Because USP5 functions as a deubiquitinase that regulates target proteins at the posttranscriptional level, the molecular mechanisms underlying USP5-mediated EMT still need to be further investigated. Altogether, these findings suggest that USP5 induces Slug expression and promotes EMT in lung cancer.

EMT confers metastatic properties on cancer cells by enhancing mobility and invasion in several cancers, including lung cancer [35]. We next examined the role of USP5 in regulating the metastasis of lung cancer cells. Indeed, knockdown of USP5 expression significantly decreased cell motility and invasion in CL1-5 cells (Fig. 3D, F). In contrast, overexpression of USP5 promoted cell migration and invasion in A549 cells (Fig. 3E, G). To investigate the role of USP5 in tumor metastasis, we established a lung metastasis mouse model by subcutaneously injecting CL1-5 cells stably expressing firefly luciferase into the left posterior flank of NOD-SCID mice. After 21 days, we evaluated lung metastasis using an IVIS imaging system. The results clearly showed that knockdown of USP5 expression in CL1-5 cells had suppressed lung metastasis at day 21 postinjection (Fig. 3H). Thus, these results indicate that USP5 drives EMT and metastasis in lung cancer.

#### USP5 promotes stemness properties by deubiquitinating β-catenin

**Fig. 4:**
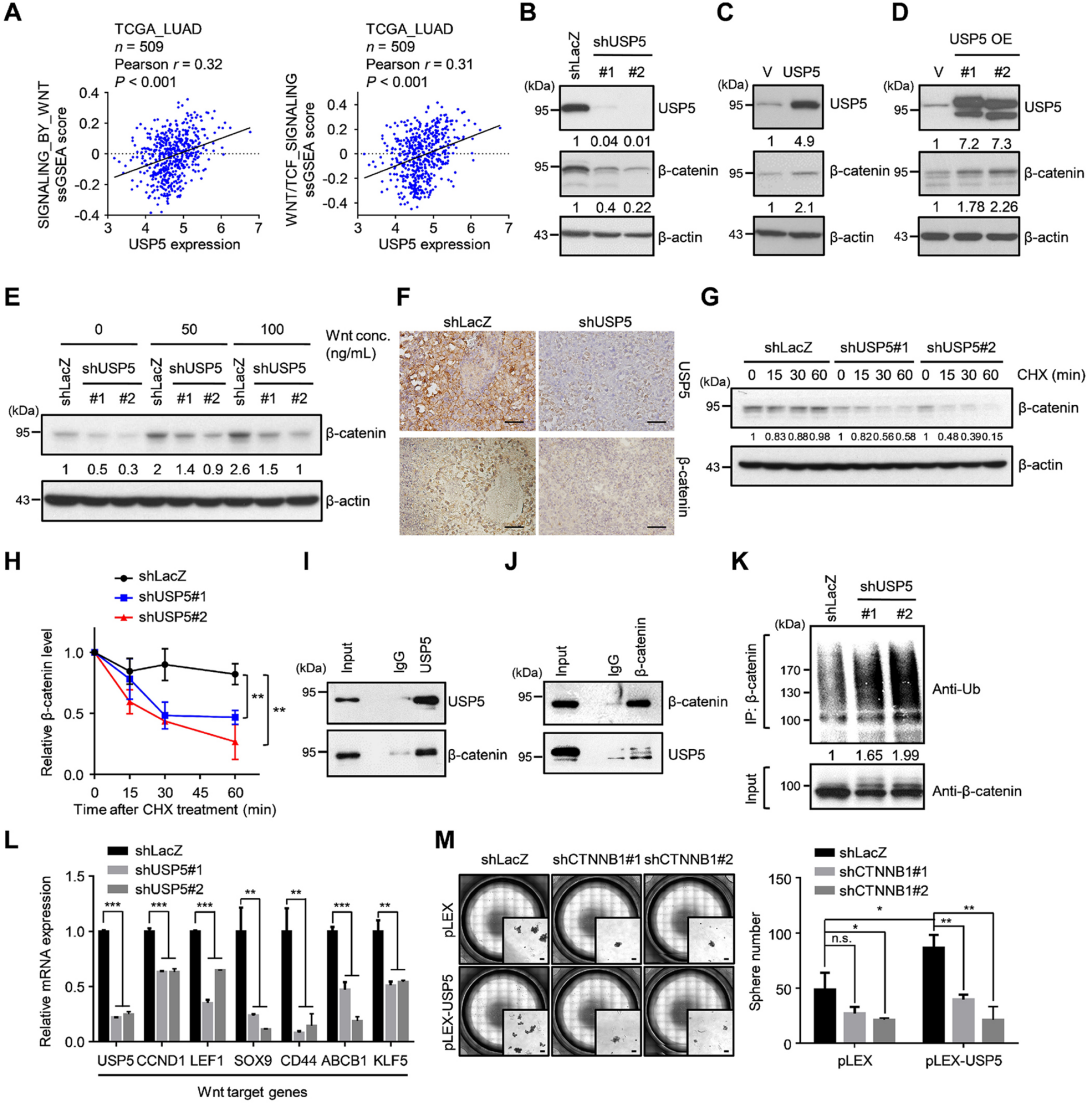
USP5 promotes Wnt/β-catenin signaling by deubiquitinating and stabilizing β-catenin. **A** Significant correlations between *USP5* expression and ssGSEA scores of the REACTOME_SIGNALING_BY_WNT and REACTOME_TCF_DEPENDENT_ SIGNALING_IN_RESPONSE_TO_WNT gene sets in the TCGA-LUAD dataset. *r*, Pearson correlation coefficient. **B–D** Protein levels of USP5 and β-catenin in CL1-5-shLacZ, shUSP5#1 and shUSP5#2 cells **(B)**, A549 cells stably expressing a vector or USP5 **(C)**, and CL1-5 cells stably expressing a vector or USP5 isoform #1 or #2 under Wnt3a treatment **(D)**. β-actin was used as an internal control. **E** CL1-5-shLacZ, shUSP5#1 and shUSP5#2 cells treated with different concentration of Wnt3A and tested to measure the protein levels of USP5 and β-catenin. **F** Immunohistochemical staining for USP5 and β-catenin in CL1-5-shLacZ and shUSP5#2 tumor samples. Scale bar: 50 μm. **G** and **H** CL1-5-shLacZ, shUSP5#1 and shUSP5#2 cells treated with 20 µg/ml protein synthesis inhibitor cycloheximide (CHX) for 60 min. The indicated proteins were detected by western blotting **(G)**, and the results were quantified with ImageJ software **(H)**. **P < 0.01 by a two-tailed Student’s *t* test. **I** Immunoprecipitation of endogenous USP5 in CL1-5 cells with antibodies against USP5 or an IgG control antibody. The associated β-catenin protein was detected with an anti-β-catenin antibody. **J** Immunoprecipitation of endogenous β-catenin in CL1-5 cells with antibodies against β-catenin or an IgG control antibody. The associated USP5 protein was detected with an anti-USP5 antibody. **K** CL1-5-shLacZ, shUSP5#1 and shUSP5#2 cells treated with 10 µM MG132 for 6 h, followed by immunoprecipitation of ubiquitinated β-catenin proteins under denaturing conditions with anti-β-catenin antibodies. The ubiquitination of β-catenin was detected by western blotting using an anti-ubiquitin antibody. **L** The mRNA levels of Wnt target genes in CL1-5-shLacZ, shUSP5#1 and shUSP5#2 cells. **P < 0.01 and ***P < 0.001 by a two-tailed Student’s *t* test. **M** A549 cells stably expressing either pLEX or pLEX-USP5 was used for silencing β-catenin expression, and each group was cultured at 1,000 cells per well in low-attachment plates to assess sphere formation. Left panel, representative stitched brightfield images were produced using NIS-Elements software (Nikon). Scale bar: 100 μm. Right panel, quantification of sphere formation after 14 days. *P < 0.05, **P < 0.01 by a two-tailed Student’s *t* test

Based on the above results, we found that depletion of USP5 significantly suppressed stemness properties, EMT, and metastasis in lung cancer. To explore downstream targets of USP5, we performed GSEA and ssGSEA using the TCGA-LUAD dataset. Focusing on stemness-related signaling, we found that both ssGSEA and GSEA showed that USP5 expression was positively correlated with Wnt/TCF-regulated signatures (Fig. 4A and Additional file 1: Figure S5A). The Wnt/TCF signaling pathway is known to play important roles in regulating CSCs, EMT and metastasis in lung cancer [36, 37]. A previous study indicated that β-catenin, as a transcriptional coactivator in the Wnt/TCF signaling pathway, is able to convert TCF/LEF into a transcriptional activator [38]. Since β-catenin stability is tightly controlled by ubiquitination-dependent degradation, we wondered whether USP5 regulates the deubiquitination and stability of β-catenin in lung cancer. First, we found that USP5 silencing caused a reduction in β-catenin expression at the protein level but not at the mRNA level in CL1-5 cells (Fig. 4B and Additional file 1: Figure S5B) In addition, the similar results could be observed in H23, H522, and H1299 lung cancer cells (Additional file 1: Figure S5C). Overexpression of USP5 caused upregulation of β-catenin expression (Fig. 4C). USP5 has been reported to be expressed in two isoforms, and we found that both isoforms of USP5 were able to promote the expression of β-catenin (Fig. 4D). Moreover, USP5 knockdown suppressed Wnt-induced β-catenin expression (Fig. 4E). Furthermore, this correlation could be observed in tumor xenografts, with immunostaining of paraffin tissue sections showing that USP5 silencing caused a reduction in β-catenin expression (Fig. 4F). These results suggest that USP5 may regulate β-catenin expression at the posttranscriptional level.

We next evaluated the role of USP5 in regulating the stability of β-catenin. First, we used cycloheximide (CHX) to inhibit global protein synthesis and investigated the change in the β-catenin protein half-life in USP5-silenced CL1-5 cells. Interestingly, the protein half-life of β-catenin was significantly decreased after USP5 silencing (Fig. 4G, H). Moreover, a co-immunoprecipitation assay showed that USP5 interacted with β-catenin (Fig. 4I, J). To investigate the role of USP5 in the deubiquitination of β-catenin, we used MG132 to inhibit the activity of the proteasome and detected the level of ubiquitinated β-catenin. We found that an obviously increased amount of ubiquitinated β-catenin could be detected in USP5-silenced CL1-5 cells compared to control cells (Fig. 4K). Finally, consistent with the clinical relevance of USP5 and the Wnt/TCF signaling signatures, we also found that the expression of several Wnt downstream target genes was significantly suppressed in USP5-silenced cells (Fig. 4L). Collectively, these data suggest that USP5 deubiquitinates β-catenin, promotes β-catenin stability, and subsequently promotes Wnt signaling pathway activity in lung cancer cells.

To confirm the role of β-catenin in USP5-mediated stemness, we silenced the β-catenin expression in USP5-overexpressing A549 cells and determined the sphere-forming ability (Additional file 1: Figure S5D). Our results revealed that USP5-overexpressing A549 cells exhibited increased sphere formation, and depletion of β-catenin expression abolished this phenomenon (Fig. 4M). Taken together, these results suggest that β-catenin plays a critical role in USP5-mediated stemness.

### Inhibition of USP5 by small molecules enhances β-catenin ubiquitination and suppresses stemness properties and cell motility

**Fig. 5:**
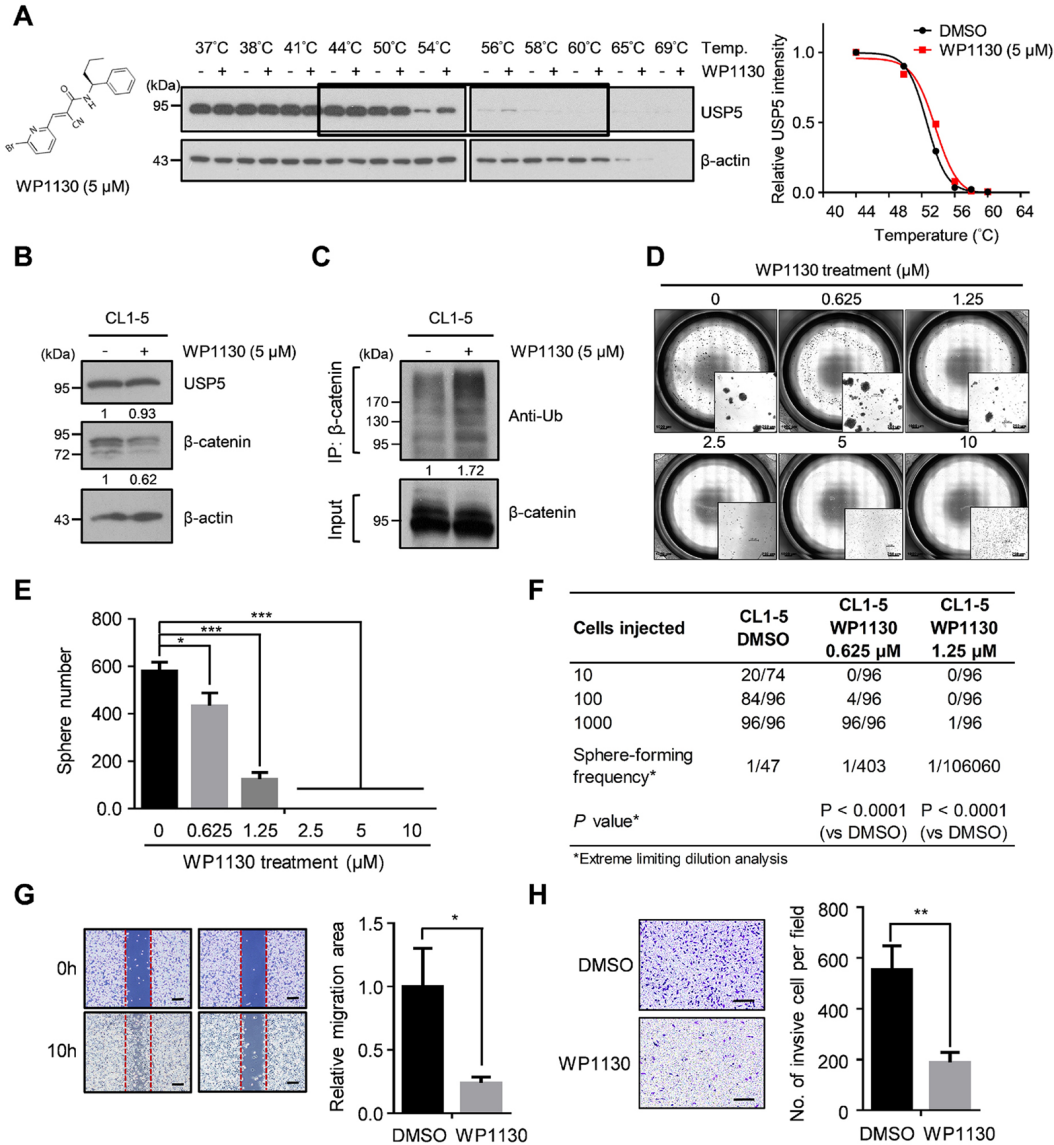
Targeting USP5 with WP1130 suppresses β-catenin expression, stemness and cell motility. **A** CETSA-based determination of interaction between WP1130 and USP5. Chemical structure of WP1130 (left). The results of immunoblotting of USP5 thermal aggregation curves of WP1130 at 5µM compared to DMSO control sample (middle). β-actin was used as an internal control. Representative images are shown. The band intensities of USP5 were normalized with respect to the intensity at 44 °C (right). **B** The protein levels of USP5 and β-catenin in CL1-5 cells treated with either DMSO (control) or 5 µM WP1130. β-actin was used as an internal control. **C** DMSO- or WP1130-treated CL1-5 cells were treated with 10 µM MG132 for 6 h and then subjected to immunoprecipitation under denaturing conditions using anti-β-catenin antibodies. The ubiquitination of β-catenin was detected by western blotting using an anti-ubiquitin antibody. **D **and **E** DMSO- and WP1130-treated CL1-5 cells were cultured at 10,000 cells per well in low-attachment plates to assess sphere formation. Representative stitched brightfield images were produced using NIS-Elements software (Nikon) **(D)**, and spheres were quantified after 14 days, **P < 0.01 by a two-tailed Student’s *t* test **(E)**. **F** The frequency of sphere-initiating cells. DMSO- and WP1130-treated CL1-5 cells were plated in low-attachment 96-well plates for a limiting-dilution assay. The percentage of wells with spheres was measured. The frequency and *P*-value were calculated using ELDA software. **G** Wound healing analysis of DMSO- and WP1130-treated CL1-5 cells at 10 h. Scale bar: 200 μm. **H** Transwell invasion analysis of DMSO- and WP1130-treated CL1-5 cells. Scale bar: 200 μm. In all bar plots, *P*-values were determined by a two-tailed Student’s *t* test

Based on the abovementioned results, we hypothesized that targeting USP5 may be beneficial in treating lung cancer patients. To test this hypothesis, we used WP1130, a selective deubiquitinase inhibitor (USP5, UCH-L1, USP9x and USP14), as the agent in our study [39]. CETSA is a newly developed and label-free assay that could be used to study drug binding to target proteins in cells based on the ability of ligand binding to enhance the thermal stabilization of target proteins [40]. To determine whether WP1130 binds to USP5, CETSA was firstly employed in CL1-5 cell lysates. We observed that WP1130 treatment significantly increased the thermal stability of USP5. The *T*_m_ value increased from 52.6 °C (control) to 53.6 °C (WP1130-treated), suggesting that WP1130 induced thermal stabilization of USP5 (Fig. 5A). An experiment showed that WP1130 treatment led to β-catenin degradation in both CL1-5 cells and LIJ cells, a primary cell line isolated from a patient with NSCLC (Fig. 5B and Additional file 1: Figure S6A). Moreover, WP1130 treatment enhanced the polyubiquitination of β-catenin in both CL1-5 cells and LIJ cells (Fig. 5C and Additional file 1: Figure S6B). Since WP1130 showed inhibitory effects on USP5 and other 3 DUBs, we used another small compound, Vialinin A, that is able to inhibit the enzyme activities of USP5 and USP4 in further tests [41]. Similarly, treatment with Vialinin A also caused downregulation of β-catenin expression in CL1-5 cells (Additional file 1: Figure S6C). Next, we also tested whether WP1130 treatment inhibits USP5-mediated functions in lung cancer. First, WP1130 treatment significantly suppressed sphere formation (Fig. 5D–F). Moreover, WP1130 treatment also significantly suppressed cell motility and invasion in CL1-5 cells (Fig. 5G, H). Therefore, this evidence shows that targeting USP5 using the small compound WP1130 may have therapeutic potential for lung cancer treatment.

### Lung tumors with high levels of ***USP5***, Wnt signaling score, and Stemness score display the lowest overall survival rates

**Fig. 6:**
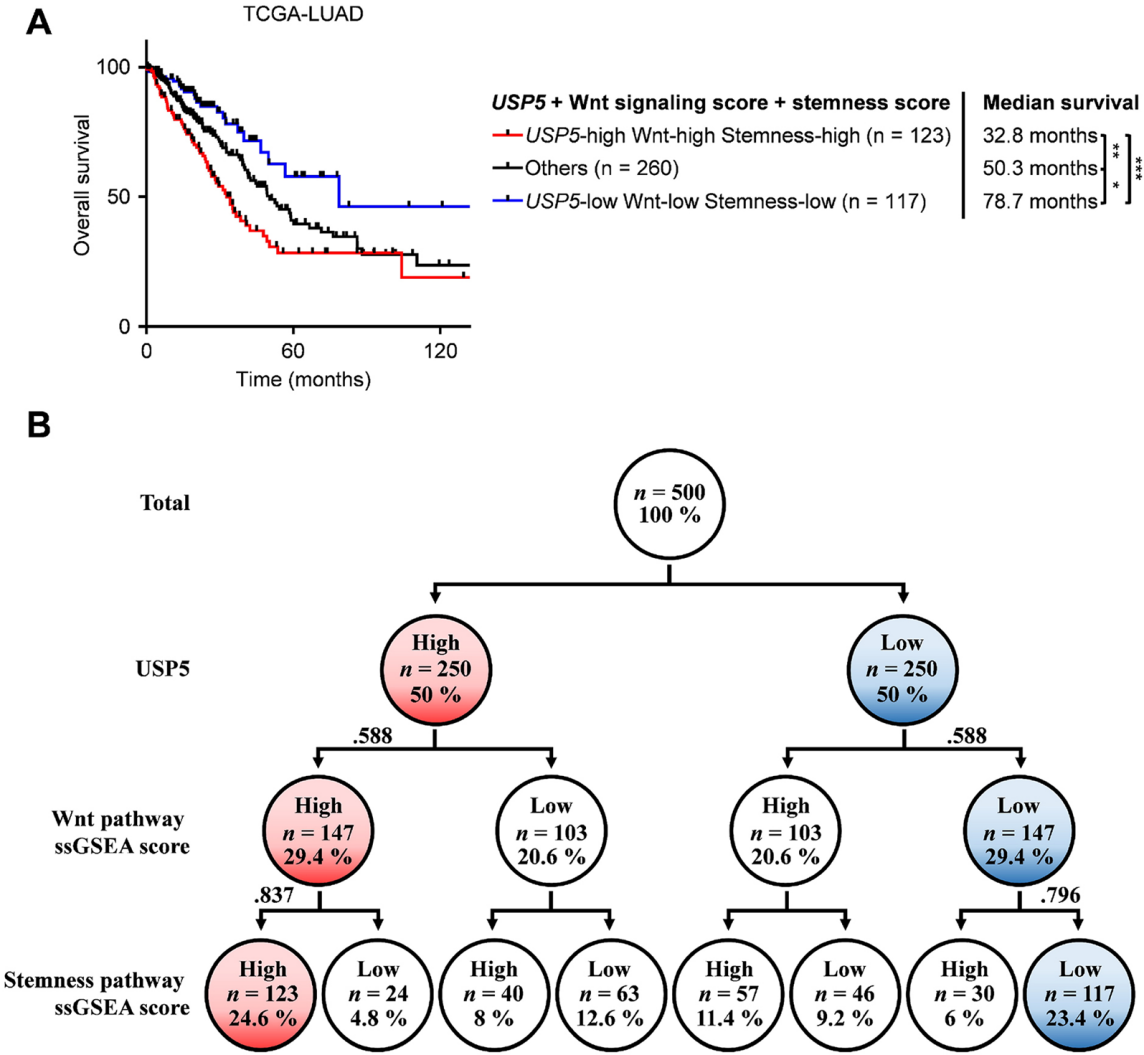
Kaplan-Meier survival curves of lung cancer patients stratified by the combined markers USP5 expression, Wnt signaling, and Stemness scores. Global gene expression data were obtained for 500 lung cancer patients in the TCGA-LUAD dataset. The ssGSEA scores for Wnt signaling and stemness were calculated with the *R/Bioconductor* package GSVA (v1.34.0) using the gene sets of REACTOME_TCF_DEPENDENT_SIGNALING_IN_ RESPONSE_TO_WNT and BENPORATH_ES_CORE_NINE, respectively. **A** The patients were divided into high and low groups for each factor using the median value as the cutoff. The combined effects of *USP5* expression, Wnt signaling, and stemness on the overall survival of lung cancer patients were analyzed. The median survival of each molecular subtype is indicated. ***P < 0.001, **P < 0.01, *P < 0.05 by the log-rank test. **B** Relative proportions of patients categorized based on *USP5* expression and Wnt signaling and Stemness scores

Our above results consistently showed that USP5 could regulate Wnt/TCF signaling and stemness properties. However, the clinical impacts remained unclear. To determine the effect of a high level of USP5, Wnt signaling, and stemness in clinical specimens, we used ssGSEA scores to determine the level of Wnt signaling (gene set: REACTOME_TCF_DEPENDENT_SIGNALING_IN_RESPONSE_TO_WNT) and stemness (gene set: BENPORATH_ES_CORE_NINE) in the TCGA-LUAD dataset. *USP5* levels, Wnt signaling scores, and Stemness scores were dichotomized into high and low groups using the median value as the cutoff. Analysis of the combined effect of USP5, Wnt signaling, and stemness on patient prognosis revealed that patients with higher USP5, Wnt signaling, and stemness levels (median survival, 32.8 months) had much poorer survival than those showing lower levels (median survival, 78.7 months) (Fig. 6A). In the cohorts analyzed in this study, 24.6% of the lung cancer cases exhibited high levels of USP5 expression/Wnt signaling/stemness, while 23.4% of the lung cancer cases had low levels, indicating that this pathway exists in patients with lung cancer (Fig. 6B). In addition, we also confirmed these findings by analyzing another cohort. Using the Rousseaux_2013 dataset, we observed similar results, with patients that had higher levels of USP5 expression, Wnt signaling, and stemness (median survival, 30 months) having much poorer survival than those with lower levels (median survival, 104 months) (Additional file 1: Figure S7A). For this cohort, 28% of the lung cancer cases exhibited high levels of USP5 expression/Wnt signaling/stemness, while 25.6% of the lung cancer cancers had low levels (Additional file 1: Figure S7B). Overall, our data suggest that high levels of USP5 expression/Wnt signaling/stemness predict poor overall survival in lung cancer.

## Discussion

**Fig. 7:**
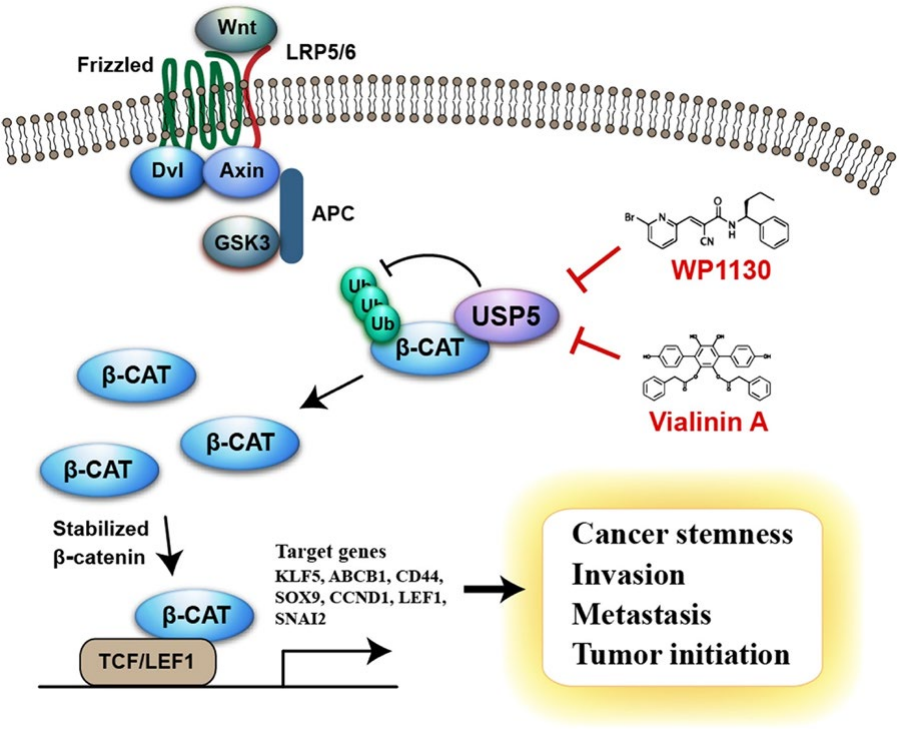
Schematic representation of targeting USP5 by WP1130 and Vialinin A for suppressing Wnt/β-catenin signaling, cancer stemness, and metastasis in lung cancer. The interaction between USP5 and β-catenin leads to the deubiquitylation of β-catenin and inhibition of proteasome-mediated degradation of β-catenin. Stabilized β-catenin then enters nucleus, activates the transcription of target genes, promotes cancer cell stemness, invasion, metastasis and tumor initiation

Lung cancer remains a highly malignant cancer and has the highest mortality rate worldwide, and metastasis is the major cause of patient death [2]. Given that mounting evidence suggests that a small proportion of stem-like cells may play an important contributory role in metastatic progression, there is an urgent need to identify critical factors that can be used as therapeutic targets in these cells [42]. Here, we identified the deubiquitinase USP5, which is highly associated with stemness-related pathways in lung cancer specimens and critical for enhancing stemness properties and metastasis in lung cancer. Mechanistically, Wnt signaling signatures were identified to be clinically related to USP5 expression, and our data showed that USP5 physically interacted with β-catenin to cause β-catenin deubiquitination and stabilization, thereby stimulating Wnt signaling. Importantly, targeting USP5 with the small molecule WP1130 was sufficient to specifically deplete lung CSCs and reduce cell motility (Fig. 7). Overall, our findings suggest that pharmacologically targeting USP5 may represent a potential strategy for targeting lung CSCs and reducing the number of deaths from lung cancer metastasis.

Our clinical analysis not only indicates the existence of a significant association between the deubiquitination process and a stemness index but also identifies USP5 as the important factor controlling stem-like properties in lung cancer. This work is also the first study to show a correlation between a stemness index and USP5 expression in lung cancer patients. Notably, our clinical analysis revealed that OTUB1 was the second DUB candidate associated with a high stemness index score and poor outcome in lung cancer patients (Additional file 1: Figure S2A, B). Intriguingly, OTUB1 is reported to stabilize SLC7A11 through CD44, which is known to be an important CSC marker in human cancers [43]. Recently, another study also provided evidence showing that OTUB1 functions as an activator of the Hippo pathway to maintain CSCs in NSCLC [44]. Altogether, these data support our clinical findings, and we have identified a new role for USP5 in regulating stemness properties in lung cancer.

The Wnt signaling pathway has long been associated with tumorigenesis, CSCs, cancer metabolism and immunity in many cancers [45]. Especially in lung cancer, Wnt signaling is a crucial pathway for driving both CSC formation and metastasis [36, 37, 46]. It is known that β-catenin is encoded by the *CTNNB1* gene, functions as the key component in canonical Wnt signaling and is tightly regulated by ubiquitin-mediated protein degradation [38]. Interestingly, mutation of the *CTNNB1* gene seems to be uncommon in lung cancer [46], highlighting the aberrant stabilization of β-catenin as a critical issue in lung cancer. Enhancement of β-catenin stability by DUBs through direct or indirect regulation has been investigated in some studies. For instance, recent data have indicated that USP9X promotes the activation of canonical Wnt signaling through deubiquitinating and stabilizing DVL2 in breast cancer cells [47]. In colorectal cancer, USP20 has been reported to deubiquitinate and stabilize β-catenin, which in turn promotes cancer cell growth, invasion and chemoresistance [48]. Similarity, USP4 also acts as an oncoprotein to regulate β-catenin deubiquitination in colorectal cancer [49]. Recently, Xue’s study revealed that knockdown of USP5 expression caused downregulation of the β-catenin protein level and induction of GSK-3β phosphorylation on Ser9 [50]. However, Ser9 phosphorylation of GSK-3β inactivates this protein and thereby increases β-catenin protein stability [51], indicating that other mechanisms control the degradation of β-catenin when USP5 is inhibited. Here, our findings provide new insights indicating that USP5 interacts with β-catenin, causes β-catenin deubiquitination, prolongs the β-catenin protein half-life, and thus increases the expression of Wnt/β-catenin downstream target genes in lung cancer.

Our study showed WP1130 inhibited cell motility and cancer stemness in lung cancer. Published evidence suggests that WP1130 is a partially selective DUB inhibitor that can inhibit the deubiquitinating activity of USP5, UCH-L1, USP9x, and USP14 [39]. Among these DUBs, USP9X was also reported to promote Wnt signaling. Thus, we could not rule out the possibility that WP1130-mediated effects were partially mediated by another DUB. In confirmation that the effects were dependent on USP5, our data revealed that treatment with Vialinin A, an inhibitor of USP5 and USP4 [41], also showed an inhibitory effect on β-catenin expression in CL1-5 cells (Additional file 1: Figure S6C). Compared to the strong clinical correlation among USP5, Wnt signaling, and stemness in the TCGA-LUAD dataset, there was no correlation among USP9X, Wnt signaling, and stemness in clinical specimens. Overall, these findings suggest that USP5 serves as an important target of these small compounds for suppression of Wnt signaling and stemness properties.

## Conclusions

In summary, our work demonstrates that USP5 promotes β-catenin stabilization and Wnt signaling activation to control cancer stemness and metastasis in lung cancer. Additionally, targeting USP5 with either WP1130 or Vialinin A suppresses USP5-mediated effects. Furthermore, patients with high levels of USP5 expression, Wnt signaling activity, and stemness activity were found to have significantly shorter overall survival than those with low levels. Altogether, this study indicates that targeting USP5 with small molecules in these patients may have beneficial effects that improve future lung cancer therapeutics.

### Supplementary Information

**Additional file 1: Table S1.** Sequences of specific paired primers used in this study. **Figure S1.** Deubiquitination-related Pathway is positively correlated with stemness and poor overall survival in TCGA-LUAD dataset. a Heat map showing 135 reactome  pathways positively correlated with stemness index mRNAsi in TCGA-LUAD dataset. The ssGSEA score of each pathway was determined using *R/Bioconductor *package *GSVA *(v1.34.0). Correlation between pathway ssGSEA score and mRNAsi was determined using Pearson correlation analysis. Pathways with the correlation coefficients larger than 0.7 were selected for the positively correlated pathways. b Univariate Cox regression analysis of ssGSEA pathway scores for overall survival in TCGA-LUAD dataset. Forest plot showing the top 10 pathways (in the 135 stemness-associated pathways) associated with poor overall survival in TCGA-LUAD dataset. HR, hazard ratios; CI, confidence intervals. **Figure S2.** *USP5 *is associated with mRNAsi and clinical outcomes in human lung cancer. a Heat map showing the correlation between 7 DUBs and stemness index mRNAsi in TCGA-LUAD dataset. Correlation was determined using Pearson correlation analysis. b, c The expression levels of *USP5 *in lung tumor samples (*n *= 91) were significantly higher than normal lung tissues (n = 65) in both GSE19188 (b) and GSE2514 © dataset. d The expression levels of *USP5 *are significantly higher in late-stage tumors (Stage III, IV; *n *= 26), compared to early-stage tumors (Stage I, II; *n *= 85) in GSE3141 dataset. e *USP5 *expression in lung cancer patients with lymph node metastasis (N1; *n *= 52) are significantly higher than those without metastasis (N0; *n *= 129) in GSE50081 dataset. *P*-value was determined by Mann-Whitney *U *test. **Figure S3.** USP5 promotes sphere formation in lung cancer. a A549 cells stably expressing either pLEX or pLEX-USP5 was cultured at 5,000 cells per well in low-attachment plates to assess sphere formation. Representative stitched brightfield images were produced using NIS-Elements software (Nikon). Scale bar: 200 μm. b Quantification of spheres generated by A549 cells stably expressing either pLEX or pLEXUSP5 after 14 days. *P < 0.05 by a two-tailed Student’s t test. **Figure S4.** USP5 correlates with *SNAI2* expression and EMT in lung cancer. A Correlation between stemness index mRNAsi and ssGSEA score of gene set SARRIO_EMT_UP in TCGA-LUAD dataset, determined using Pearson’s correlation analysis. b The mRNA levels of *SNAI2 *in CL1-5-shLacZ, shUSP5#1 and shUSP5#2 cells. ***P < 0.001 by two-tailed Student’s *t*-test. c, d The relationships between *USP5*and *SNAI2* were determined from gene expression data from GSE50081 (c) and GSE17710 (d) datasets using Pearson’s correlation analysis. **Figure S5.** Knockdown of *USP5 *suppresses β-catenin expression in lung cancer cells. a Gene set enrichment analysis reveals *USP5 *expression is positively correlated with Wnt signaling pathways using TCGA-LUAD dataset. Black bars at the bottom of the figure indicate the location of genes in each gene set. b The mRNA levels of *CTNNB1*and *GAPDH *in CL1-5-shLacZ, shUSP5#1 and shUSP5#2 cells. c The protein levels of USP5 and β-catenin in control (shLacZ) and USP5-depleted (shUSP5) lung cancer cells. β-actin was used as an internal control. d The protein levels of USP5 and β-catenin in USP5-overexpressing and control A549 cells with or without β-catenin knockdown. **Figure S6.** Targeting USP5 via small compounds suppresses β-catenin expression and enhances its ubiquitination in lung cancer cells. a The protein levels of USP5 and β-catenin in LIJ cells treating with either DMSO control or WP1130. b DMSO- or WP1130-treated LIJ cells were treated with 10 μM MG132 for 6 h and pulled down under denaturing conditions using anti-β-catenin antibodies. The ubiquitinated β-catenin was detected by western blotting using an anti-ubiquitin antibody. c The protein levels of USP5 and β-catenin in CL1-5 cells treating with either DMSO control or Vialinin A. β-actin was used as an internal control. **Figure S7.** Kaplan-Meier survival curve of lung cancer patients based on the combined markers of USP5, Wnt signaling, and stemness scores. The global gene expression data was obtained from 293 lung cancer patients in the Rousseaux_2013 dataset. The ssGSEA scores of Wnt signaling and stemness were calculated by *R/Bioconductor *package *GSVA *(v1.34.0) using gene sets of REACTOME_TCF_DEPENDENT_SIGNALING_IN_RESPONSE_TO_WNT and BENPORATH_ES_CORE_NINE, respectively. a The patients were divided into the high- and lowexpression of each factor using the median value as the cutoff. The combined effects of *USP5*, Wnt signaling, and stemness on the overall survival of lung cancer patients were analyzed. The median survival of each molecular subtype is indicated. **P < 0.01, *P< 0.05 by log-rank test. b Relative proportions of patients categorized based on *USP5 *expression, Wnt signaling score, and stemness score.

## Data Availability

All data generated or analyzed during this study are included in this published article and its supplementary information files.

## Abbreviations

CSCs: Cancer stem cells
ssGSEA: Single-sample gene set enrichment analysis
mRNAsi: mRNA expression-based stemness index
TCGA: The Cancer Genome Atlas
LUAD: Cohort of lung adenocarcinoma
USP5: Ubiquitin-specific peptidase 5
EMT: Epithelial-mesenchymal transition
DUBs: Deubiquitinating enzymes
FPKM: Fragments per kilobase of transcript per million mapped reads
FBS: Fetal bovine serum
ATCC: The American Type Culture Collection
NSCLC: Non-small-cell lung cancer
qRT-PCR: Quantitative real-time PCR
poly-HEMA: Poly-2-hydroxyethyl methacrylate
LDA: Limiting dilution analysis
ELDA: Extreme limiting dilution analysis
CETSA: Cellular thermal shift assay
NOD-SCID: Non-obese diabetic-severe combined immunodeficiency
