# Supplementary material for: Ubiquitin-specific peptidase 5 facilitates cancer stem cell-like properties in lung cancer by deubiquitinating β-catenin
Source: Cancer Cell Int. 2023 Sep 19;23:207. doi: 10.1186/s12935-023-03059-6 (PMC10510149; doi:10.1186/s12935-023-03059-6)
Supplement: Supplementary file 1 — Additional file 1: Table S1. Sequences of specific paired primers used in this study. Figure S1. Deubiquitination-related Pathway is positively correlated with stemness and poor overall survival in TCGA-LUAD dataset. a Heat map showing 135 reactome pathways positively correlated with stemness index mRNAsi in TCGA-LUAD dataset. The ssGSEA score of each pathway was determined using R/Bioconductor package GSVA (v1.34.0). Correlation between pathway ssGSEA score and mRNAsi was determined using Pearson correlation analysis. Pathways with the correlation coefficients larger than 0.7 were selected for the positively correlated pathways. b Univariate Cox regression analysis of ssGSEA pathway scores for overall survival in TCGA-LUAD dataset. Forest plot showing the top 10 pathways (in the 135 stemness-associated pathways) associated with poor overall survival in TCGA-LUAD dataset. HR, hazard ratios; CI, confidence intervals. Figure S2. USP5 is associated with mRNAsi and clinical outcomes in human lung cancer. a Heat map showing the correlation between 7 DUBs and stemness index mRNAsi in TCGA-LUAD dataset. Correlation was determined using Pearson correlation analysis. b, c The expression levels of USP5 in lung tumor samples (n = 91) were significantly higher than normal lung tissues (n = 65) in both GSE19188 (b) and GSE2514 © dataset. d The expression levels of USP5 are significantly higher in late-stage tumors (Stage III, IV; n = 26), compared to early-stage tumors (Stage I, II; n = 85) in GSE3141 dataset. e USP5 expression in lung cancer patients with lymph node metastasis (N1; n = 52) are significantly higher than those without metastasis (N0; n = 129) in GSE50081 dataset. P-value was determined by Mann-Whitney U test. Figure S3. USP5 promotes sphere formation in lung cancer. a A549 cells stably expressing either pLEX or pLEX-USP5 was cultured at 5,000 cells per well in low-attachment plates to assess sphere formation. Representative stitched brightfield images were produc [file 12935_2023_3059_MOESM1_ESM.pdf]

Supplementary Table 1. Sequences of specific paired primers used in this study

Gene	Primer	Sequence (5' to 3')
CTNNB1	Forward	CTTGGTTCACCAGTGGATTC
	Reverse	GAGTCCCAAGGAGACCTTCC
GAPDH	Forward	TGAAGGTCGGAGTCAACGGATT
	Reverse	CCTGGAAGATGGTGATGGGATT
USP5	Forward	AGACCACACGATTTGCCTCA
	Reverse	AGCTCCTCTGGCATCTCGAT
TBP	Forward	CACGAACCACGGCACTGATT
	Reverse	TTTTCTTGCTGCCAGTCTGGAC
SNAI2	Forward	ACAGCGAACTGGACACACATAC
	Reverse	TCTCTGGTTGTGGTATGACAGG
CCND1	Forward	CCGCTGGCCATGAACTACCT
	Reverse	ACGAAGGTCTGCGCGTGTT
LEF1	Forward	GCCACGGACGAGATGATCC
	Reverse	TGTCTGGCCACCTCGTGTC
SOX9	Forward	AGCGAACGCACATCAAGAC
	Reverse	CTGTAGGCGATCTGTTGGGG
CD44	Forward	TGCCGCTTTGCAGGTGTAT
	Reverse	GGCCTCCGTCCGAGAGA
ABCB1	Forward	GCCTTCATCGAGTCACTGCC
	Reverse	GGCTGTCTAACAAGGGCACG
KLF5	Forward	CGCATCCACTACTGCGATTA
	Reverse	CAGCCTTCCCAGGTACACTT
POU5F1	Forward	CTTGCTGCAGAAGTGGGTGGAGGAA
	Reverse	CTGCAGTGTGGGTTTCGGGCA
NANOG	Forward	AATACCTCAGCCTCCAGCAGATG
	Reverse	TGCGTCACACCATTGCTATTCTTC
ABCG2	Forward	TCATCAGCCTCGATATTC
	Reverse	GGCCCGTGGAACATAAGTCTT

Supplementary Figure 1

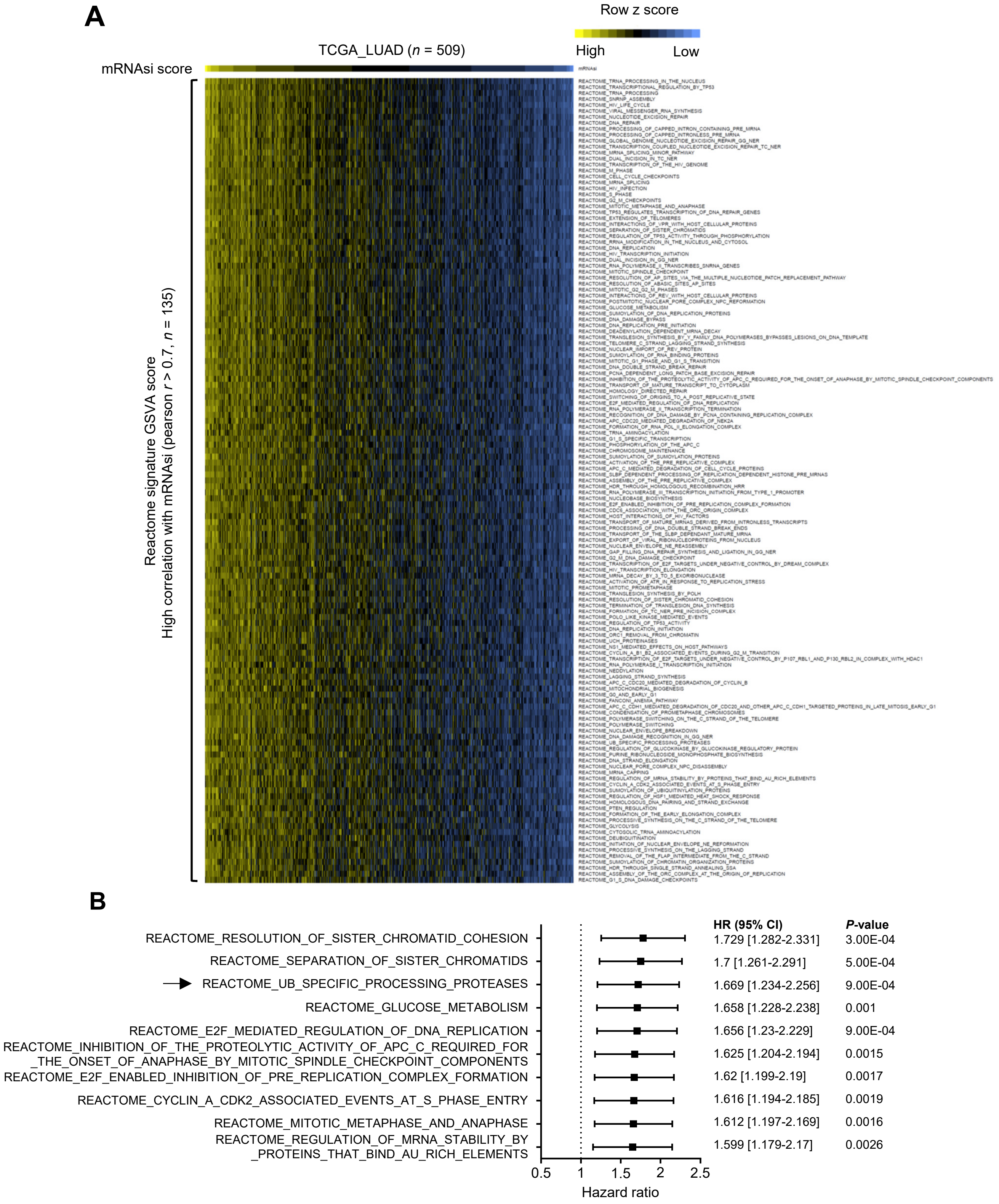

Supplementary Figure 1. Deubiquitination-related Pathway is positively correlated with stemness and poor overall survival in TCGA-LUAD dataset. a Heat map showing 135 reactome pathways positively correlated with stemness index mRNAsi in TCGA-LUAD dataset. The ssGSEA score of each pathway was determined using *R/Bioconductor* package *GSVA* (v1.34.0). Correlation between pathway ssGSEA score and mRNAsi was determined using Pearson correlation analysis. Pathways with the correlation coefficients larger than 0.7 were selected for the positively correlated pathways. **b** Univariate Cox regression analysis of ssGSEA pathway scores for overall survival in TCGA-LUAD dataset. Forest plot showing the top 10 pathways (in the 135 stemness-associated pathways) associated with poor overall survival in TCGA-LUAD dataset. HR, hazard ratios; CI, confidence intervals.

Supplementary Figure 2

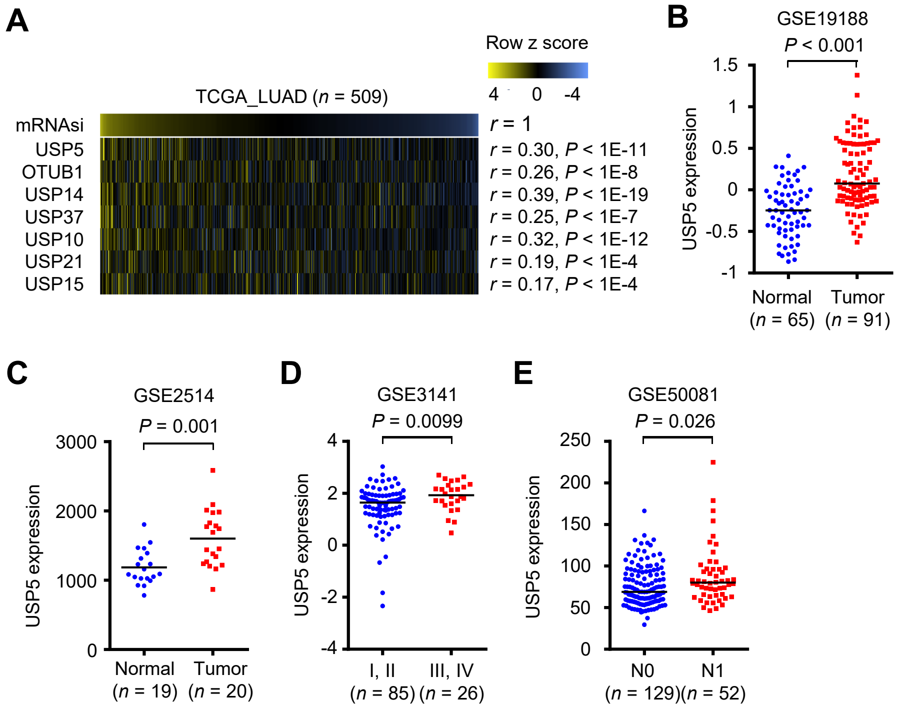

Supplementary Figure 2. *USP5* is associated with mRNAsi and clinical outcomes in human lung cancer. a Heat map showing the correlation between 7 DUBs and stemness index mRNAsi in TCGA-LUAD dataset. Correlation was determined using Pearson correlation analysis. **b, c** The expression levels of *USP5* in lung tumor samples ($n = 91$) were significantly higher than normal lung tissues ($n = 65$) in both GSE19188 **(b)** and GSE2514 © dataset. **d** The expression levels of *USP5* are significantly higher in late-stage tumors (Stage III, IV; $n = 26$), compared to early-stage tumors (Stage I, II; $n = 85$) in GSE3141 dataset. **e** *USP5* expression in lung cancer patients with lymph node metastasis (N1; $n = 52$) are significantly higher than those without metastasis (N0; $n = 129$) in GSE50081 dataset. *P*-value was determined by Mann-Whitney *U* test.

Supplementary Figure 3

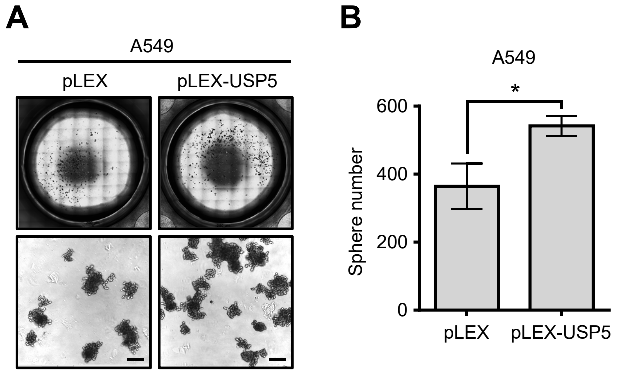

Supplementary Figure 3. USP5 promotes sphere formation in lung cancer. a A549 cells stably expressing either pLEX or pLEX-USP5 was cultured at 5,000 cells per well in low-attachment plates to assess sphere formation. Representative stitched brightfield images were produced using NIS-Elements software (Nikon). Scale bar: 200 µm. **b** Quantification of spheres generated by A549 cells stably expressing either pLEX or pLEX-USP5 after 14 days. *P < 0.05 by a two-tailed Student's t test.

Supplementary Figure 4

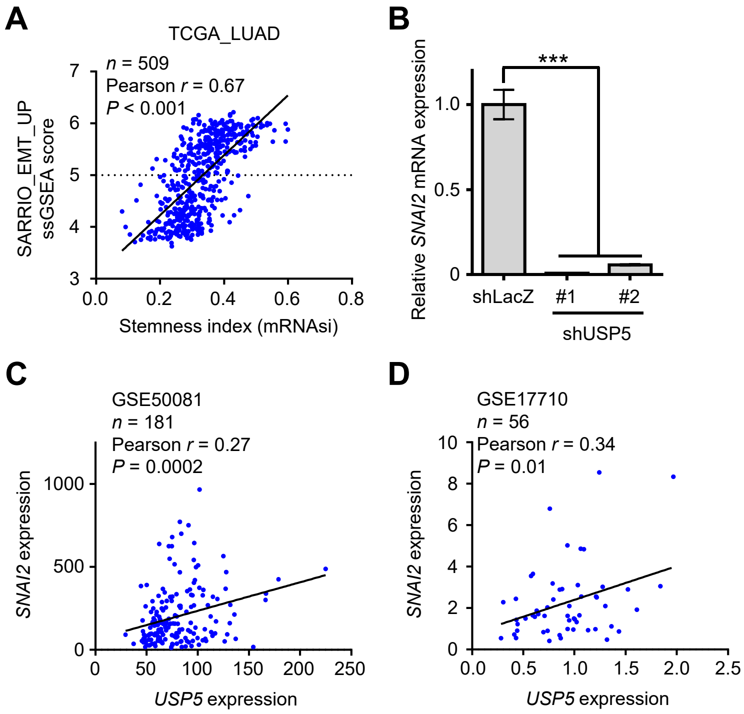

Supplementary Figure 4. a Correlation between stemness index mRNAsi and ssGSEA score of gene set SARRIO_EMT_UP in TCGA-LUAD dataset, determined using Pearson's correlation analysis. **b** The mRNA levels of *SNAI2* in CL1-5-shLacZ, shUSP5#1 and shUSP5#2 cells. ***$P < 0.001$ by two-tailed Student's *t*-test. **c, d** The relationships between *USP5* and *SNAI2* were determined from gene expression data from GSE50081 **(c)** and GSE17710 **(d)** datasets using Pearson's correlation analysis.

Supplementary Figure 5

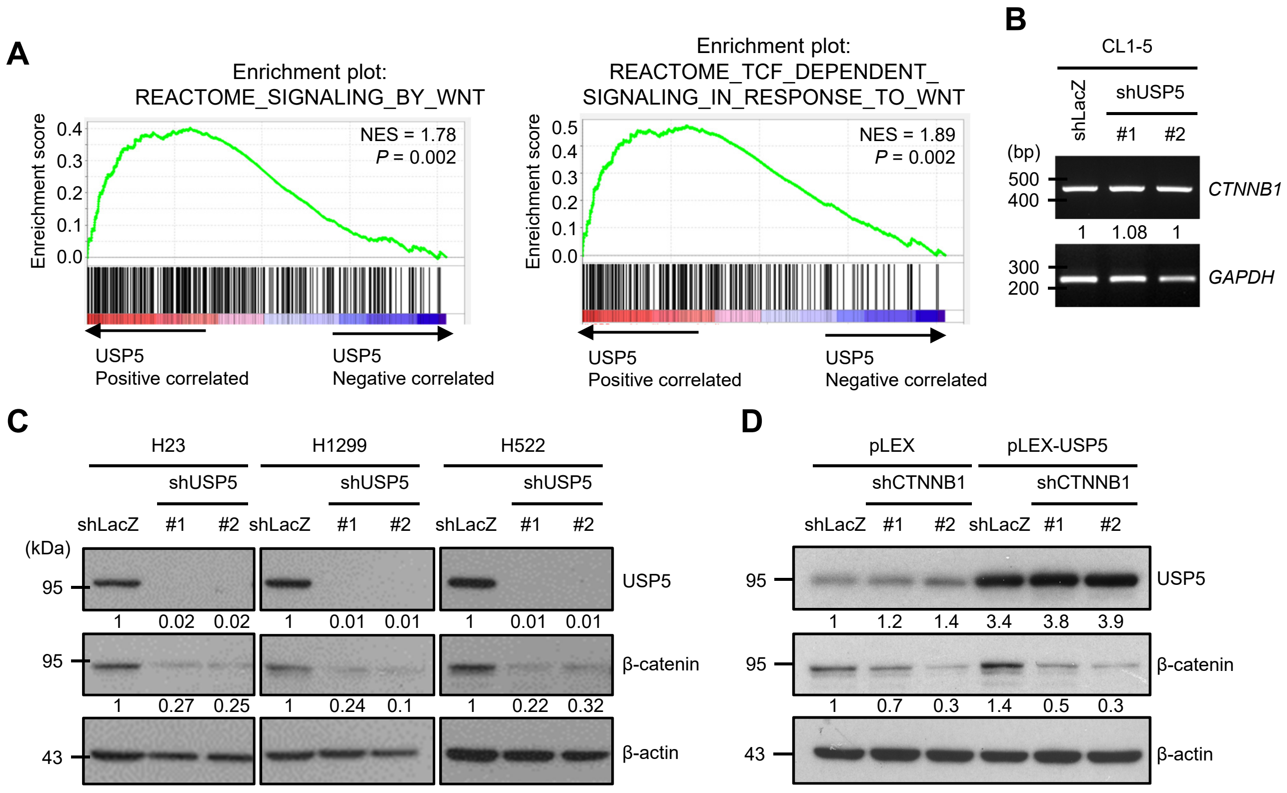

Supplementary Figure 5. Knockdown of *USP5* suppresses β-catenin expression in lung cancer cells. a Gene set enrichment analysis reveals *USP5* expression is positively correlated with Wnt signaling pathways using TCGA-LUAD dataset. Black bars at the bottom of the figure indicate the location of genes in each gene set. **b** The mRNA levels of *CTNNB1* and *GAPDH* in CL1-5-shLacZ, shUSP5#1 and shUSP5#2 cells. **c** The protein levels of USP5 and β-catenin in control (shLacZ) and USP5-depleted (shUSP5) lung cancer cells. β-actin was used as an internal control. **d** The protein levels of USP5 and β-catenin in USP5-overexpressing and control A549 cells with or without β-catenin knockdown.

Supplementary Figure 6

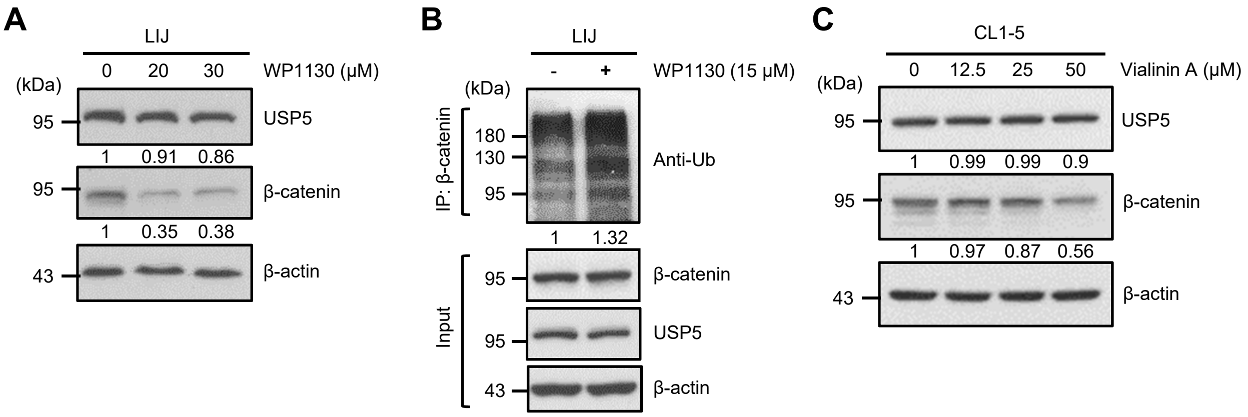

Supplementary Figure 6. Targeting USP5 via small compounds suppresses β-catenin expression and enhances its ubiquitination in lung cancer cells. a The protein levels of USP5 and β-catenin in LIJ cells treating with either DMSO control or WP1130. **b** DMSO- or WP1130-treated LIJ cells were treated with 10 μM MG132 for 6 h and pulled down under denaturing conditions using anti-β-catenin antibodies. The ubiquitinated β-catenin was detected by western blotting using an anti-ubiquitin antibody. **c** The protein levels of USP5 and β-catenin in CL1-5 cells treating with either DMSO control or Vialinin A. β-actin was used as an internal control.

Supplementary Figure 7

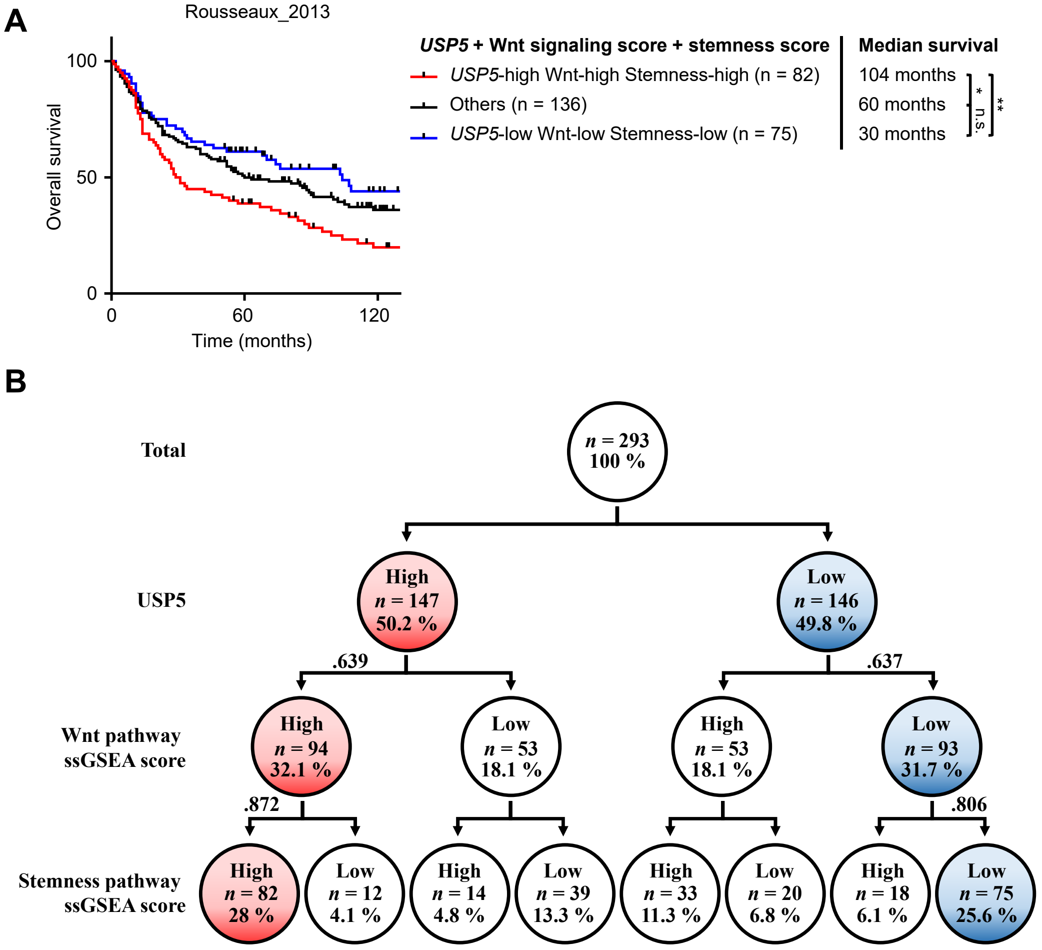

Supplementary Figure 7. Kaplan-Meier survival curve of lung cancer patients based on the combined markers of USP5, Wnt signaling, and stemness scores. The global gene expression data was obtained from 293 lung cancer patients in the Rousseaux_2013 dataset. The ssGSEA scores of Wnt signaling and stemness were calculated by *R/Bioconductor* package *GSVA* (v1.34.0) using gene sets of REACTOME_TCF_DEPENDENT_SIGNALING_IN_RESPONSE_TO_WNT and BENPORATH_ES_CORE_NINE, respectively. **a** The patients were divided into the high- and low-expression of each factor using the median value as the cutoff. The combined effects of *USP5*, Wnt signaling, and stemness on the overall survival of lung cancer patients were analyzed. The median survival of each molecular subtype is indicated. $**P < 0.01$, $*P < 0.05$ by log-rank test. **b** Relative proportions of patients categorized based on *USP5* expression, Wnt signaling score, and stemness score.
